# Multifunctional macrophage mimetic nanoplatform modulates vascular and epithelial double gut barriers to alleviate ulcerative colitis

**DOI:** 10.7150/thno.118236

**Published:** 2026-01-01

**Authors:** Weijian Cheng, Yixi Zhu, Miaoxizi Luo, Xiao Wang, Quanlong Chen, Siyao Li, Jing Xian, Meng Xiao, Licheng Liu, Yuanyuan Wang, Chaomei Fu, Ruibing Wang, Qian Cheng, Jinming Zhang

**Affiliations:** 1 State Key Laboratory of Southwestern Chinese Medicine Resources, School of Pharmacy, Chengdu University of Traditional Chinese Medicine, Chengdu, 611137, China.; 2 State Key Laboratory of Quality Research in Chinese Medicine, Institute of Chinese Medical Sciences, University of Macau, Taipa, Macau SAR, 999078, China.; 3 Faculty of Pharmacy and Food, Southwest Minzu University, Chengdu & Key Laboratory of Research and Application of Ethnic Medicine Processing and Preparation on the Qinghai Tibet Plateau, 610225, China.

**Keywords:** ulcerative colitis, self-assembly, biomimetic nano-formulations, intestinal epithelial barrier, gut vascular barrier

## Abstract

**Rationale:** In ulcerative colitis (UC), microbial products or metabolites, coupled with inflammatory stimuli, result in simultaneous damage to both the intestinal epithelial barrier (IEB) and gut vascular barrier (GVB). Current UC treatments usually focus on modulating IEB, whereas GVB—which critically regulates the translocation of gut microbiota and metabolites into systemic circulation—has been largely overlooked. Here, we developed a facile, biomimetic strategy to engineer anti-inflammatory berberine/magnolol self-assembled nanoparticles (BM NPs) using macrophage membrane camouflage, enabling targeted UC accumulation and dual restoration of both the IEB and GVB.

**Methods:** BM NPs employing macrophage membranes to camouflage mimetic nanoplatform. The mimetic nanoplatform on targeting capacity of inflamed intestinal epithelial cells, M1/M2 polarization, macrophage and intestinal epithelial cell inflammatory factors, and vascular endothelial cell migration and tube-forming were evaluated *in vitro*. Furthermore, its therapeutic efficacy was assessed in a mice UC model, demonstrating significant reductions in bacterial translocation, restoration of both the IEB and GVB, and modulation of the inflammatory immune microenvironment.

**Results:** The biomimetic nanoplatform demonstrates superior targeting specificity and prolonged retention in inflamed intestinal epithelium and vascular tissues. Macrophage membranes achieve GVB repair by mechanical traction and physical adsorption of inflammatory factors. Besides, efficient delivery of the loaded anti-inflammatory drugs also achieves the repair of the IEB. GVB repair effectively prevents systemic dissemination of gut-derived microbes and their metabolites, thereby attenuating UC-induced inflammatory cascades. Collectively, this approach significantly ameliorates colonic pathology in UC.

**Conclusion:** Our study proposes the synergistic repair of IEB and GVB through the mechanical traction and physical adsorption of macrophage membranes, assisted by anti-inflammatory components, which provide new insights as well as a new paradigm for the treatment of UC.

## Introduction

Ulcerative colitis (UC) is a chronic inflammatory disease that invades the rectum and colon, and its incidence rate is rising worldwide 1. UC is characterized by complex pathogenesis, closely related to the overactivation of immune cells in the inflammatory immune microenvironment, abnormal intestinal flora and disruption of the intestinal barrier 2-4. Conventionally, to restore intestinal epithelial barrier (IEB) and regulate gut flora, employing 5-aminosalicylic acid, glucocorticoids, immunosuppressive agents and probiotics, are the most used strategies for UC treatment 5, 6. However, the current therapeutic outcomes are not satisfactory, neither in continuously alleviating the disease symptoms nor in reducing the recurrence rate 7. Accumulating evidence suggests that dysfunction of gut vascular barrier (GVB) correlates with several clinical pathologies including UC and extra-intestinal diseases, such as non-alcoholic fatty liver disease and central nervous system disease 8. GVB plays a major dual role in the body: on the one hand, it controls the migration of antigens and intestinal microorganisms from the gut to the bloodstream, and on the other hand, it hinders the entry into the circulation of bacteria that has broken through the IEB 9-11. Until recently, the leakage of GVB, as the deepest layer of the gut barrier, has been observed in the state of colitis, so that the bacterial toxins in the gut cavity would enter the blood circulation, causing systemic inflammation 12. Therefore, the progression of UC disrupts the GVB, leading to the systemic dissemination of hazardous substances and inflammatory responses, which aggravates UC and even some systemic diseases. The dual intestinal barrier disruptions ultimately lead to dysregulation of the immune microenvironment, such as the evolution of macrophages to a pro-inflammatory phenotype, and the production of inflammatory cytokines, chemokines, and ROS by macrophages to continuously trigger immune responses 13, 14. At the same time, the disruption of the intestinal barrier leads to the transfer of intestinal commensal flora and pathogenic microorganisms, which disrupts the balance of the intestinal bacterial community and leads to intestinal infections with harmful bacteria 15, 16. However, there is still a gap in UC therapeutic research that can simultaneously the repair of the dual intestinal barriers, including IEB and GVB.

In recent years, the advantages of natural active ingredient combination for UC suppression have well been demonstrated. Similarly, berberine (Ber) and magnolol (Mag), derived from a renowned TCM classical prescription Huanglian-Houpo Decoction, have exhibited multi-target therapeutic potential in UC involving in anti-inflammatory, antioxidant, and intestinal epithelial barrier repair. Specifically, Ber suppresses NF-κB/MAPK signaling and NLRP3 inflammasome activation, modulates Th17/Treg balance, enhances tight junction proteins, and restores gut microbiota homeostasis. Mag potentiates these effects by activating PPARγ and the cholinergic anti-inflammatory pathway 17. Their synergy amplifies suppression of pro-inflammatory cytokines, attenuates oxidative stress, and mitigates the dysbiosis-barrier dysfunction-inflammation axis. The combination of Ber and Mag represents a promising strategy for adjunct UC therapy, particularly in refractory cases requiring alternatives to conventional anti-inflammatory agent or immunomodulators. Inspired by their anti-UC benefits, an appropriate delivery approach for Ber and Mag combination is required. Indeed, accurately locating the inflamed area in the colon is challenging due to the complex gastrointestinal environment. Currently, most of the previous studies still focus on the oral delivery of colon-targeted micro/nanoparticulate systems as the appealing and promising approaches 18. Various colon-targeted systems, with enteric, mucosal adhesive, controlled/sustained drug release properties, are well recognized to favor the accumulation and the raised of residence time in the inflamed intestinal area 19, 20. Nevertheless, these oral delivery systems cannot efficiently cross the diseased mucosal barrier, let alone repair GVB.

Biomimetic nano-formulations, especially formulated by the membrane camouflage strategy, have attracted increasing attention owe to the excellent biocompatibility, extending the half-life of nanoparticles in systemic circulation and the intrinsic chemotactic capacity to inflammatory sites 21, 22. As the important component of immune cells, macrophages play a critical role in the occurrence and progression of inflammation-associated diseases, in which to maintain homeostasis and modulate immune responses simultaneously 23. Therefore, macrophage-biomimetic nanoplatforms well inherit the pro-inflammatory factors scavenging activity of macrophages and exhibit the excellent application potential in inflammation-associated diseases, such as the treatment of tumor, atherosclerosis, and rheumatoid arthritis 24-28. Interestingly, studies have shown that macrophages can repair vascular injuries by secreting vascular endothelial growth factors (VEGF) and physical adhesion to achieve traction 29-31. Macrophage membranes (MM) would inherit the potential to repair vascular injury by physical adhesion of macrophages to achieve traction 32. In addition, researchers have recently discovered that MM can effectively capture and neutralize different types of cytokines, such as IFN-γ, IL-6, and TNF-α, due to the presence of receptors that have a particularly high affinity for pro-inflammatory cytokines 33-35. In contrast to the clinical use of monoclonal antibodies such as anti-TNF-α and anti-IL-1β antibodies with the clearance effects of single inflammatory factors, MM exhibits more superior anti-inflammatory efficacy against multiple cytokines. Therefore, a novel design idea was put forward to formulate a MM-camouflaged nanosystem for UC treatment via the intravenous administration. In that way, the MM-camouflaged system could be actively delivered and colonized into the damaged GVB site, and then infiltrate into the colitis site, to restore the disordered GVB and IEB simultaneously.

**Scheme 1:**
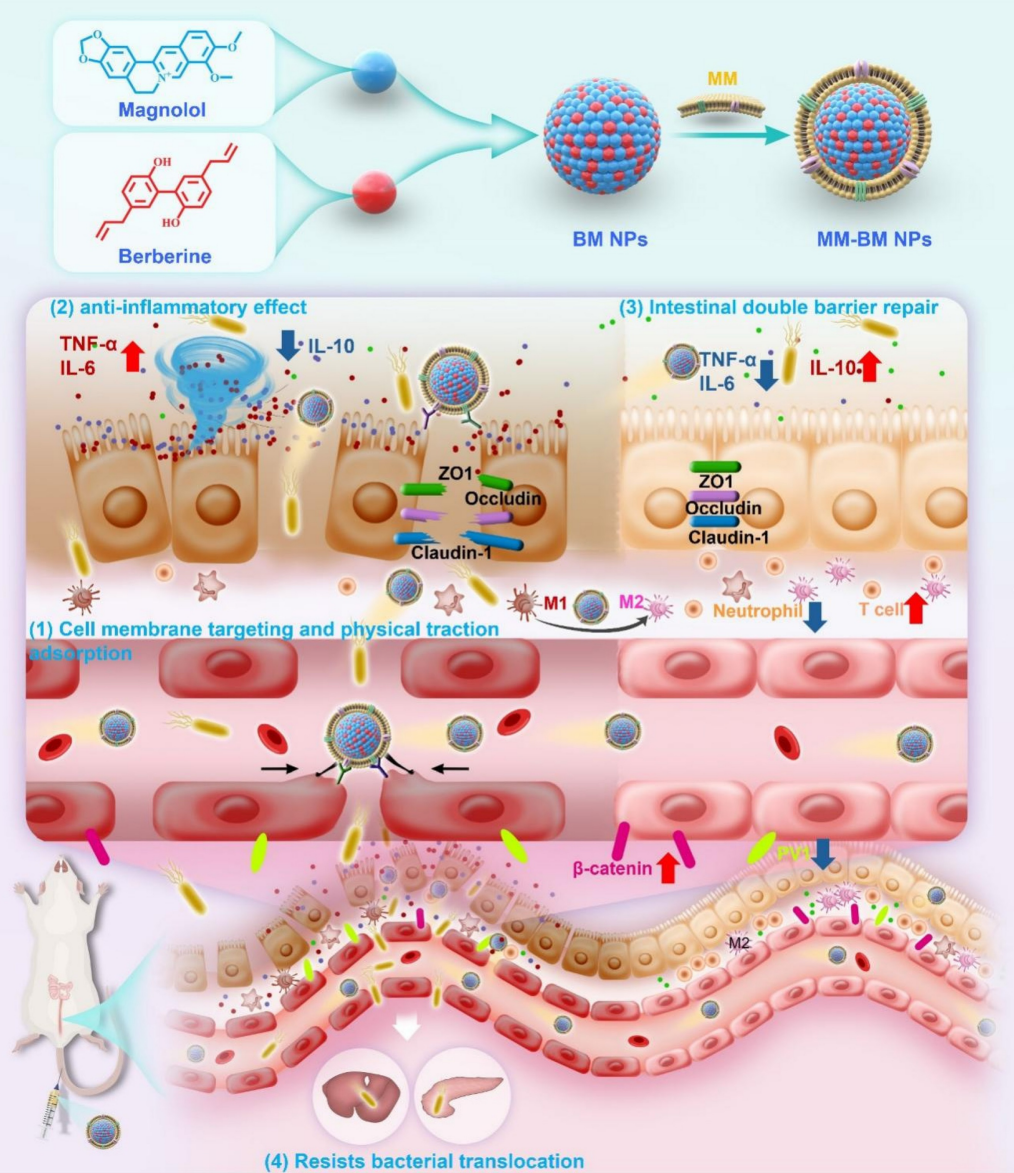
Schematic illustration of the anti-UC mechanisms of MM-BM NPs by modulating vascular and epithelial double gut barriers.

In this study, the supramolecular self-assembly nanosystem of Ber and Mag (BM NPs) was spontaneously formulated to synergistically repair the fragile gut microenvironment in colitis, in which the self-assembly mechanism was well revealed. As a proof-of-concept, we further performed the MM-camouflaged nano-system (MM-BM NPs) to actively accumulate at the damaged gut blood vessels as the GVB repairing additive, relying on the physical adsorption of MM against inflammatory factors to produce a mechanical traction effect (Scheme 1). Based on the tail intravenous injection on UC mice, MM-BM NPs avoided clearance by the reticuloendothelial system and significantly enhanced accumulation in the inflamed colon and vascular system of UC mice. It also repairs the function of the double intestinal barrier, which in turn restores the balance of the intestinal flora, resulting in a well-regulated inflammatory microenvironment and ultimately a significant improvement in the pathology of the inflammatory colon. Therefore, this work reveals the mechanism by which cell membrane biomimetic nanoparticles can multifunctionally regulate UC diseases and provides a new perspective for other inflammatory diseases.

## Materials and Methods

### Materials

Berberine, magnolol (98%) were purchased from Chengdu Alfa Biotechnology Co., Ltd. (Chengdu, China). Coumarin-6 (C6) was provided by Aladdin Reagent Company (Shanghai, China). Dextran sulfate Sodium (DSS, MW 40 kDa) was purchased from Seebio Biotechnology Co., Ltd. (Shanghai, China). The lipophilic carbocyanine dye 1,1'-dioctadecyl-tetramethylindotricarbocyanine iodide (DiR, >95%) and 1,1'-dioctadecyl-3,3,3',3'-tetramethylindocarbocyanine perchlorate (Dil, >95%) were purchased from Dalian Meilun Biotechnology Co., Ltd. (Dalian, China). 4′ ,6-Diamidino-2-phenylindole (DAPI) was obtained from Sigma-Aldrich Co., LLC. The interleukin-6 (IL-6), interleukin-10 (IL-10), the tumor necrosis factor-α (TNF-α) kit were supplied by Multi Science (Lianke) Biotech Co., Ltd. (Hangzhou, China). F4/80, CD86, CD206, CD3. CD4, CD8, CD45, CD11b, Ly6G antibodies were supplied by BioLegend Co., Ltd. (San Diego, CA, USA). Tetrahydrofuran (THF) was purchased from Chengdu Cologne Chemicals Co., Ltd. (Chengdu, China). Dimethyl sulfoxide (DMSO) was provided by Macklin Biochemical Co., Ltd. (Shanghai, China).

### Preparation of BM NPs

The self-assembly BM NPs were prepared by an antisolvent precipitation method. Briefly, 13.3 mM Mag THF solution was adjusted to pH 7-7.5 with sodium bicarbonate solution (0.1 M) and then slowly added dropwise to 500 μM Ber solution (molar ratio, 4:1) with heating and stirring to evaporate 2 h. The resulting solution was filtered through a microporous membrane (0.8 μm) to obtain the self-assembled BM NPs.

### Molecular dynamics (MD)

The simulation box was constructed and randomly filled with Ber and Mag molecules respectively to form a complex simulation system. MD simulation was carried out under constant temperature and pressure and periodic boundary conditions using Gromacs 2018.4 program. The energy of the complex system is minimized by the steepest method to eliminate the close contact between atoms. The MD simulation of the complex system was performed at 50 ns, and the conformation was saved every 10 ps. The visualization of the simulation results was completed by Gromacs embedded program and VMD.

### Isothermal titration calorimetry (ITC)

ITC measurements were performed on MICROCAL VP-ITC (Malvern, UK). Titrate the Ber solution (5 mM) into the Mag solution (0.25 mM). An aqueous solution of Ber (5 mM) was injected into deionized water as a benchmark. The theoretical titration curve was fitted with Nano Analyze software (NanoAnalyze v3.10.0).

### Preparation of MM-BM NPs

1×10^8^ RAW 264.7 cells were suspended with 1.5 mL of ice-cold TM buffer (10 mM Tris, 1 mM MgCl_2_, pH 7.4), and then cell membranes were damaged using an ultrasonic cell crusher (power 100 W). Then 0.5 mL of 1 M sucrose solution was added to the cell solution and centrifuged at 2000 g, 4 °C for 10 min to collect them in supernatant. It was centrifuged twice at 3000 g, 4 °C for 30 min to collect the cell membrane precipitate and resuspended with TM buffer solution and 1M sucrose solution in a ratio of 3:1 and then centrifuged again according to 3000 g, 4 °C for 30 min. After centrifugation, cell membranes were resuspended with distilled water or PBS solution. Finally, macrophage membrane nanovesicles were obtained after sonication of macrophage membrane solution probes for 3 min.

The different ratios of macrophage membrane suspension and BM NPs were mixed and sonicated in an ice-water bath for 3 min. The unloaded vesicles in the mixed suspension were removed by centrifugation at 2000 g at 4 °C and washed with cold PBS. MM-BM NPs with uniform particle size was obtained through 0.8 μm microporous membrane filtration.

The calculated formula of drug encapsulation efficiency (EE) is as follows:

EE= (W_Ber/Mag loaded in NPs_)/W_Ber/Mag total feeding_ × 100%

W_Ber/Mag loaded in NPs_ indicated the drug amount determined in BM NPs, W_Ber/Mag total feeding_ indicated the total feeding drug amount.

### Characterization of MM-BM NPs

The size distribution, ζ-potential, and polydispersity index (PDI) were measured by dynamic Light scattering (DLS) with a Particle Analyzer Lite sizer 500 (Anton Paar, Austria). The morphology of BM NPs and MM-BM NPs were determined by a Transmission electron microscopy (TEM, JEM 1200X, JEOL, Japan). Fourier transform infrared spectroscopy (FT-IR) (IR Tracer-100, Shimadzu, Japan), and ultraviolet-visible (UV-Vis) (PerkinElmer Lambda1050, USA) were used to confirm the structure of the composite units and the self-assembly mechanism.

### Characterization of MM proteins

Membrane proteins were extracted from macrophages, macrophage cell membranes, BM NPs, and MM-BM NPs and further determined by sodium dodecyl sulphate-polyacrylamide gel electrophoresis (SDS-PAGE) assay. The CD11b and Integrin α4 were determined by Western blot on RAW 264.7, MM, BM NPs, and MM-BM NPs.

### Co-localization of MM-BM NPs

C6 is encapsulated into the core of BM NPs by the preparation method of BM NPs. The MM was incubated with Dil dye for 20 min and then centrifuged at 3000 g for 5 min to collect the precipitate. Then, the Dil labeled MM is overlaid on the C6-labeled BM NPs by the MM-BM NPs preparation method. Dye-labeled MM-BM NPs was added to cultured RAW 264.7 cells. After incubation for 4 h, the cells were washed three times with PBS, fixed with tissue fixatives for 10 min, and then the cell nucleus was stained with DAPI. Cells were observed by confocal microscopy.

### Drug release profile *in vitro*

2 mL of BM, BM NPs, and MM-BM NPs (Ber concentration of 800 µg/mL and Mag concentration of 200 µg/mL) were added to the dialysis bag (MWCO: 3500 Da), respectively. At the same time, Tween 80 was added to the release medium at a concentration of 1% (w/v) to increase the solubility of Ber and Mag in the release medium. Then, dialysis bags containing different preparations were immersed in 30 mL of PBS release medium (pH 7.4) and placed on a shaker at 37 °C for 100 rpm. 1 mL of release medium was taken at preset times (0.5, 1, 2, 4, 6, 8, 10, 12, 24, 48 h) points and 1 mL of new release medium was added. Concentrations of Ber and Mag were determined by HPLC.

### Migration assay

HUVECs cells induced with TNF-α (20 ng/mL) were inoculated in 6-well plates (1×10^5^ cells/well) and then scratched in parallel with a pipette tip. Then, different preparation groups (BM group, BM NPs group, MM group, and MM-BM NPs group) were treated for 12 h, and the images were observed under a microscope (Eclipse Ti2, Nikon; Tokyo, Japan). Analysis with ImageJ software.

HUVECs cells induced with TNF-α were cultured with serum-free medium in transwell chambers and incubated in the lower 20% serum-containing medium for 24 h for transwell migration assays. After removing cells that were transwells with a cotton swab, the membrane was fixed with 70% ethanol and stained with crystal violet. Images of migrated cells were taken with a microscope. Analysis with ImageJ software.

### Tubule formation assay

HUVECs cells induced with TNF-α were inoculated into 96-well culture plates coated with growth factor-reducing gelsolin, respectively. Each group was treated with different media (BM group, BM NPs group, MM group, and MM-BM NPs group) for 8 h. HUVECs cells were treated with complete medium for 8 h as a control group. The morphology of cell tubules was observed by microscopy and the total length of branches was quantified by ImageJ software.

### Cellular uptake *in vitro*

NCM 460 cells were inoculated into confocal petri dishes and cultured in a constant temperature incubator for 12 h. Lipopolysaccharide (LPS) (1 μg/mL)-induced NCM 460 cells were inoculated into confocal petri dishes and cultured in a constant temperature incubator for 12 h. Then, C6, C6@BM NPs, and C6@MM-BM NPs were added and cultured for 4 h, respectively. At the end of the incubation, the cells were washed three times with cold PBS, fixed in 4% paraformaldehyde solution, and the nuclei were observed by DAPI staining. (CLSM; TCS SP8 SR; Leica, Weztlar, Germany).

NCM 460 cells were inoculated in 12-well plates and cultured for 12 h. LPS (1 μg/mL)-induced NCM 460 cells were inoculated in 12-well plates and cultured for 12 h. Then the cells were incubated with different formulations (complete medium, free C6, C6@BM NPs, and C6@MM-BM NPs) for 4 h. Complete medium was used as a control group. After incubation, cells were rinsed 3 times with cold PBS and then resuspended by centrifugation before flow cytometer (FACSCanto II, BD, USA) analysis.

### Cell viability assay

NCM 460 cells were inoculated into 96-well plates (8000 cells/well) and cultured overnight. RAW 264.7 cells were inoculated into 96-well plates (8000 cells/well) and cultured overnight. The medium was replaced with media containing different agents (BM, BM NPs, and MM-BM NPs). The total content of Ber and Mag in these preparations was at a concentration of 1-60 μM. After 24 h of treatment, the medium was replaced with fresh medium. The cells were incubated with 10 μL of CCK-8 reagents (Biyuntian, Shanghai, China) at 37 ℃ for 2 h. Finally, cell viability was calculated by measuring absorbance at 450 nm with an enzyme marker.

### *In vitro* anti-inflammatory effects

NCM 460 cells were inoculated in 12-well plates (1×10^5^ cells/well) and cultured overnight. BM, BM NPs, and MM-BM NPs (a total content of 5, 10, or 20 μM of Ber and Mag) were co-incubated with LPS (1 μg/mL) for 24 h then the cell supernatant in each well was collected. The levels of TNF-α, IL-6, and IL-10 in the cell supernatants were detected using an ELISA kit (MultiScience, China). The anti-inflammatory effects of various formulations on RAW 264.7 cells stimulated with LPS (500 ng/mL) were investigated. The contents of TNF-α, IL-6, and IL-10 in cell supernatant were determined by ELISA kit (MultiScience, China).

### *In vitro* macrophage polarization regulation

RAW 264.7 cells were inoculated on a 12-well plate (1×10^6^ cells/well) and cultured overnight. BM, BM NPs, and MM-BM NPs (Ber 4 μM, Mag 16 μM) were co-cultured with LPS (100 ng/mL) for 24 h. Expression levels of the M1 macrophage marker, CD86, and the M2 macrophage marker, CD206, were detected by flow cytometry.

### Animal experiment feeding

Male ICR mice (22-25 g) were obtained from SPF Biotechnology Co., Ltd. (Beijing, China). All *in vivo* experiments were conducted under the guidelines approved by the Institutional Animal Care and Use Committee of Chengdu University of Traditional Chinese Medicine, maintaining ethical standards and animal welfare standards (Approval Number: 2024100). It complied with the National Law on the Use of Laboratory Animals in China.

### Biodistribution in colitis mice

The distribution of the different formulations in the gastrointestinal tract after intravenous injection was imaged using the *in vivo* imaging system IVIS (PerkinElmer). The fluorescent probe DiR was loaded in self-assembly NPs to label both BM NPs and MM-BM NPs, based on the similar preparation approaches as-above. The free DiR, DiR@BM NPs, or DiR@MM-BM NPs (containing the equivalent amount of DiR at 0.5 mg/kg) was injected intravenously into normal mice or UC mice induced by continuous feeding of 3% DSS. Imaging was performed with IVIS (IVIS Spectrum, PerkinElmer, USA) at different time points of 3, 6, 12, and 24 h of intravenous administration. Meanwhile, mice colonic at 3, 12, and 24 h points were collected and imaged using an IVIS system to record the distribution of fluorescent signals.

To evaluate the colonic tissue penetration of MM-BM NPs, different C6 formulations including free C6, C6@BM NPs, and C6@MM-BM NPs (equivalent to C6 concentration of 5 mg/kg) were injected intravenously into DSS-induced UC mice. After 12 h post-injection, the colon tissues were collected and cut into 5 µm thick sections. Colonic tissue sections were stained with DAPI at 4 °C and then observed under a fluorescence microscope.

To evaluate the macrophage recruitment and blood vessel adhesion of MM-BM NPs in colonic tissues, free AMCA, AMCA@BM NPs, and AMCA@MM-BM NPs (equivalent to AMCA concentration of 5 mg/kg) were injected intravenously into DSS-induced UC mice. After 12 h post-injection, colons were collected and cut into 5 μm thick sections. Colonic tissue sections were stained with DAPI and CD31 at 4 °C. Fluorescent images were subsequently observed under a fluorescence microscope.

### Anti-UC effects in colitis mice

The mice were randomly divided into five groups: control group, Model group, BM group (free Ber + free Mag), BM NPs group, and MM-BM NPs group. Except for the control group, mice in other groups were fed drinking 3% DSS to establish the UC model. Only saline was injected intravenously to control and UC model mice. The other BM formulations (BM group, BM NPs group, MM group, and MM-BM NPs) were given to UC mice with 2.5 mg/kg Ber and 10 mg/kg Mag by intravenous injection. The body weights, stool consistency, and the presence of fecal blood of the mice were recorded daily during the experimental period to calculate the disease activity index (DAI). On day 11, these mice were euthanized. Subsequently, colonic tissues were collected, photographed and the length of the colon was recorded.

### Histological examination, immunohistochemistry (IHC) and immunofluorescence staining (IF) assay

Colon tissues were fixed with 4% paraformaldehyde, paraffin-embedded, and cut into 4 μm sections and stained with hematoxylin-eosin (H&E) and periodic acid Schiff (PAS). Major organs of mice (heart, liver, spleen, lungs, and kidneys) were stained with H&E for histopathological analysis.

The expressions of Occludin, Claudin-1, ZO-1, PV1, and β-catenin in colon tissues were detected by IHC. First, paraffin-embedded sections of colon tissue were treated with 3% hydrogen peroxide solution. The sections were then sequentially incubated with primary and secondary antibodies, followed by incubation with appropriate horseradish peroxidase solution. After sealing, images were obtained using a fluorescence microscope (Nikon Eclipse C1, Japan) and fluorescence intensity was measured using ImageJ software.

Immunofluorescence staining was performed to detect the expression of F4/80, CD86, CD206, CD31, PV1, and DAPI in colon tissues. The colon tissues were embedded in sections and incubated with primary and secondary antibodies at 4 ℃. Subsequently, the sections were stained with DAPI to show cell nuclei. Fluorescence images were captured using an inverted fluorescence microscope (Leica, Wetzlar, Germany).

### Laser speckle imaging

Male ICR mice were fasted for 12 h before surgery. Mice were anesthetized using an inhalation anesthesia machine with an isoflurane concentration of 3 ~ 4% and an anesthesia maintenance concentration of 1 ~ 2%. A laser scatter imaging system (RFLSI ZW, Shenzhen, China) was used to assess blood leakage.

### Tissue flow cytometry

The colon tissue was digested and extracted for 30 min. The tissue is centrifuged at 600 g for 20 min. Single cell suspensions were then collected. After rinsing the single cell suspensions with PBS, for the detection of macrophages they were stained with F4/80, CD86, and CD206 for 30 min at 4 °C. The single cell suspensions were stained with CD3, CD8, and CD4 for 30 min. For the detection of T cells CD3, CD8, and CD4 were used and stained at 4 °C for 30 min. To detect neutrophils CD45, CD11b, and Ly6G were used and stained at 4 °C for 30 min. All cells were detected using an FCM. Data was analyzed using FlowJo software.

### Western blotting

Colon tissue was pre-added to a protease inhibitor and cleaved on ice for 30 min with a 10-fold cleavage buffer. The sample was added to a 5-fold reduced protein load buffer and denatured in a boiling water bath for 15 min. The protein is transferred to the PVDF membrane by SDS-PAGE electrophoresis for further immune response. The membrane was combined with P38 (1:1000), NF-κB (1:1000), TLR4 (1:1000), or beta-tubulin (1:2000). After incubation overnight, they were treated with HRP goat anti-mice (1:500). Then ECL (Enhanced chemiluminescence) was used to detect protein expression in different colon tissues. Quantification using ImageJ software.

### Cytokines in colon tissue

BCA kit (Biosharp, China) was used to quantify the protein content of colon tissue. Subsequently, expression of TNF-α, IL-6, and IL-10 in colonic tissues using ELISA kit (MultiScience, China).

### 16s RNA gene sequencing analysis

Total fecal DNA from each group was extracted and used for PCR amplification of the V4 hypervariable region of 16S rDNA (primers: 515F (GTGCCAGCMGCCGCGGGTAA) and 806R (GGACTACNNGGGTATCTAAT)). Sequencing on the NovaSeq 6000 platform.

### Statistical analysis

Results were expressed as mean ± standard deviation (SD) and analyzed using GraphPad Prism 8.0 software. Statistical analyses were performed using unpaired t-tests or one-way ANOVA. p < for 0.05 indicated statistically significant differences.

## Results and Discussion

### Preparation and characterization of MM-BM NPs

**Figure 1:**
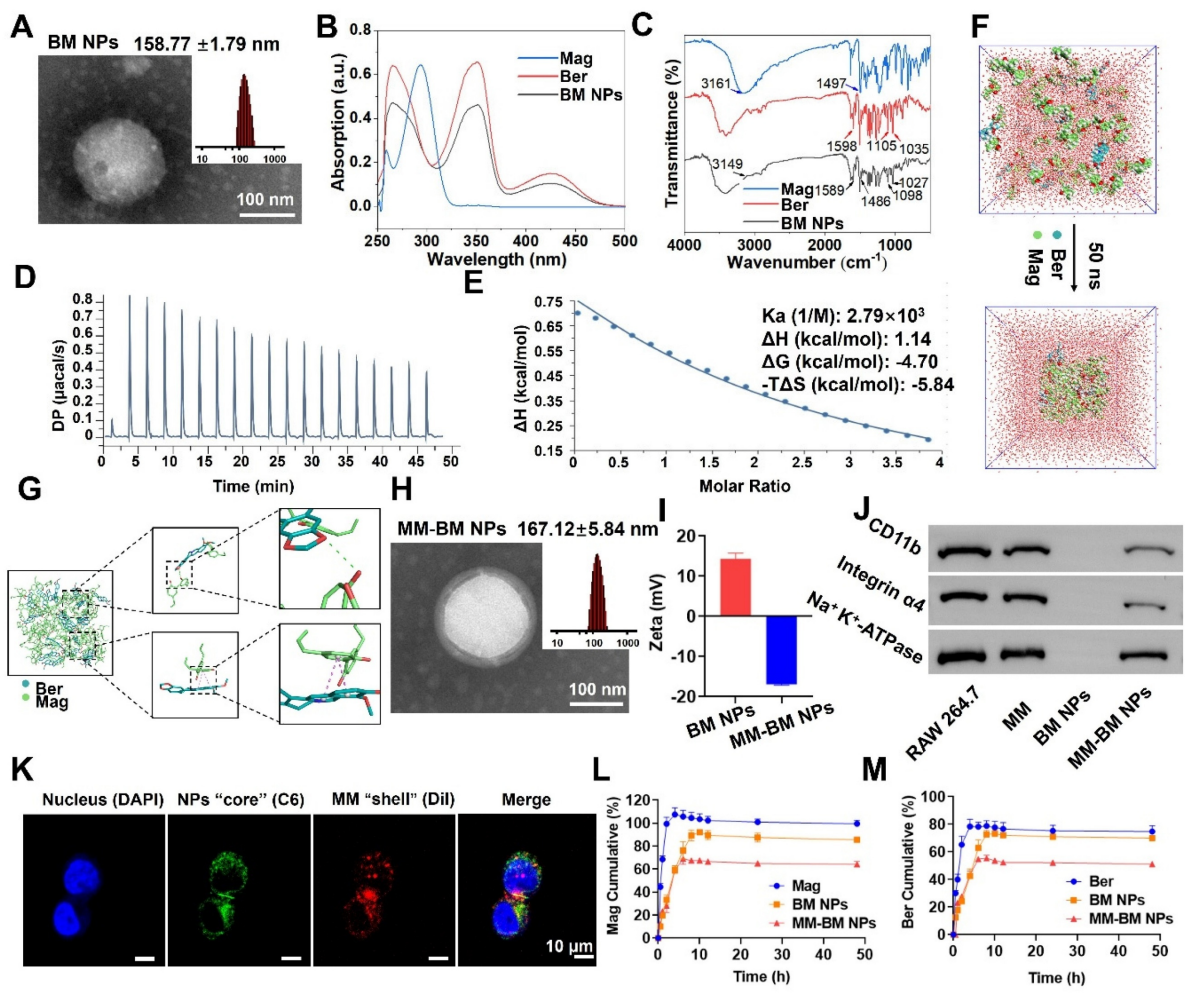
Characteristics, self-assembly identification and mechanism of action of MM-BM NPs. (A) The size distribution and TEM images of BM NPs. (B) UV-Vis spectroscopy of Ber, Mag, and BM NPs. (C) FT-IR of Ber, Mag, and BM NPs. (D) ITC data of BM NPs. (E) Fitted curves and thermodynamic parameters of the titration of BM NPs. (F) Structural changes of the BM NPs system 50 ns during the simulation (G) Molecular interaction of the BM NPs system. (H) The size distribution and TEM images of MM-BM NPs. (I) Zeta potential of BM NPs and MM-BM NPs. (J) Western blot results of CD11b and Integrin α4 in RAW 264.7 cell, MM, BM NPs, and MM-BM NPs, respectively. (K) CLSM images of the colocalization of the nucleus (DAPI), BM NPs “core” (C6) and MM “shell” (Dil) (scale bar = 10 μm). Release of Ber (L)/Mag (M), BM NPs, and MM-BM NPs *in vitro* (n = 3).

As shown in Figure 1A, TEM images showed that the BM NPs possessed a uniform spherical structure with the average size of 158.77 ± 1.79 nm measured by DLS, which was consistent with the TEM results. The formation of BM NPs was confirmed using UV-vis spectrometry in the range 250-500 nm. As shown in Figure 1B, due to the strong UV absorption of Ber, BM NPs exhibited the similar absorption spectra with Ber. However, due to the interaction among groups of Ber and Mag molecules, the maximum absorption peak wavelength of BM NPs exhibited a shift compared to free Ber. The UV spectrum of Ber displayed absorption peaks at 265 nm, 350 nm, and 425 nm, whereas BM NPs showed corresponding peaks at 267 nm, 353 nm, and 422 nm. The blue-shifted peak may be attributed to the altered energy level difference between ground and excited states after molecular self-assembly. The observed red shift suggests a potential expansion of the conjugated system, likely induced by the π-π interaction between benzene ring-rich Ber and Mag.

The FT-IR results also demonstrated the electrostatic attraction and π-π superposition between unsaturated cycloalkanes in Ber and phenolic hydroxyls in Mag. As shown in Figure 1C, the characteristic absorption bands of BM NPs are basically the same as those of Ber and Mag. In BM NPs, the C-N bond of Ber shifted from 1598 cm^-1^ to 1589 cm^-1^, while the peak width of the O-H bond of Mag increased and shifted to a lower frequency from 3161 cm^-1^ to 3149 cm^-1^, suggesting that the quaternary ammonium ions of Ber and the phenolic hydroxyls of Mag successfully self-assembled to form intermolecular hydrogen bonds. In the BM NPs, the alicyclic ether and aromatic vibrational bands of Ber move from 1035^-1^ to 1027 cm^-1^ and 1105^-1^ to 1098 cm^-1^, respectively, whereas the aromatic vibrational band of Mag moves from 1497^-1^ to 1486 cm^-1^. The results indicated that the formation of hydrogen bonds between Ber and Mag or π-π conjugation effect.

The ITC results revealed that the interaction between Ber and Mag follows an exothermic thermodynamic process (Figure 1D). As shown in Figure 1E, the formation of self-assembly of BM NPs is a spontaneous process. MD simulations showed that BM NPs forming a two-dimensional structure mainly through π-π stacking and hydrophobic interactions, where π-π stacking and hydrogen bonding lead to the formation of a dense and stable spatial spherical structure (Figure 1F-G, and S1). These results collectively indicate that Ber and Mag spontaneously self-assemble into nanoparticles driven by hydrophobic interactions and π-π stacking, with the final nanosphere structure maintained through hydrogen bonding and π-π stacking interactions. TEM imaging and DLS analysis confirmed successful membrane encapsulation, as demonstrated by the clearly visible surface bilayer and increased hydrodynamic diameter (167.12 ± 5.84 nm) of MM-BM nanoparticles (Figure 1H). Zeta potential measurements showed that while BM NPs exhibited strong positive surface charge, MM coating reversed this to negative potential (Figure 1I), a characteristic favorable for systemic circulation. Both MM-BM NPs and BM NPs demonstrated excellent colloidal stability, with only minimal size increases during storage. SDS-PAGE analysis confirmed effective retention of most macrophage membrane proteins in MM-BM NPs (Figure S2). Western blotting further verified the preservation of two key functional proteins (integrin α4 and CD11b) known to mediate targeting of inflamed endothelial cells in UC (Figure 1J). Successful membrane coating was additionally confirmed by confocal laser scanning microscopy through colocalization of C6 and Dil fluorescence signals (Figure 1K). HPLC measurements revealed excellent drug encapsulation efficiencies of 83.36% for Ber and 97.63% for Mag in MM-BM NPs. *In vitro* release studies demonstrated superior controlled release properties of MM-BM NPs compared to both BM NPs and free Ber/Mag (Figures 1L-M), suggesting minimal drug leakage during systemic circulation and enhanced delivery efficiency.

### Gut vascular barrier restoration of MM-BM NPs

**Figure 2:**
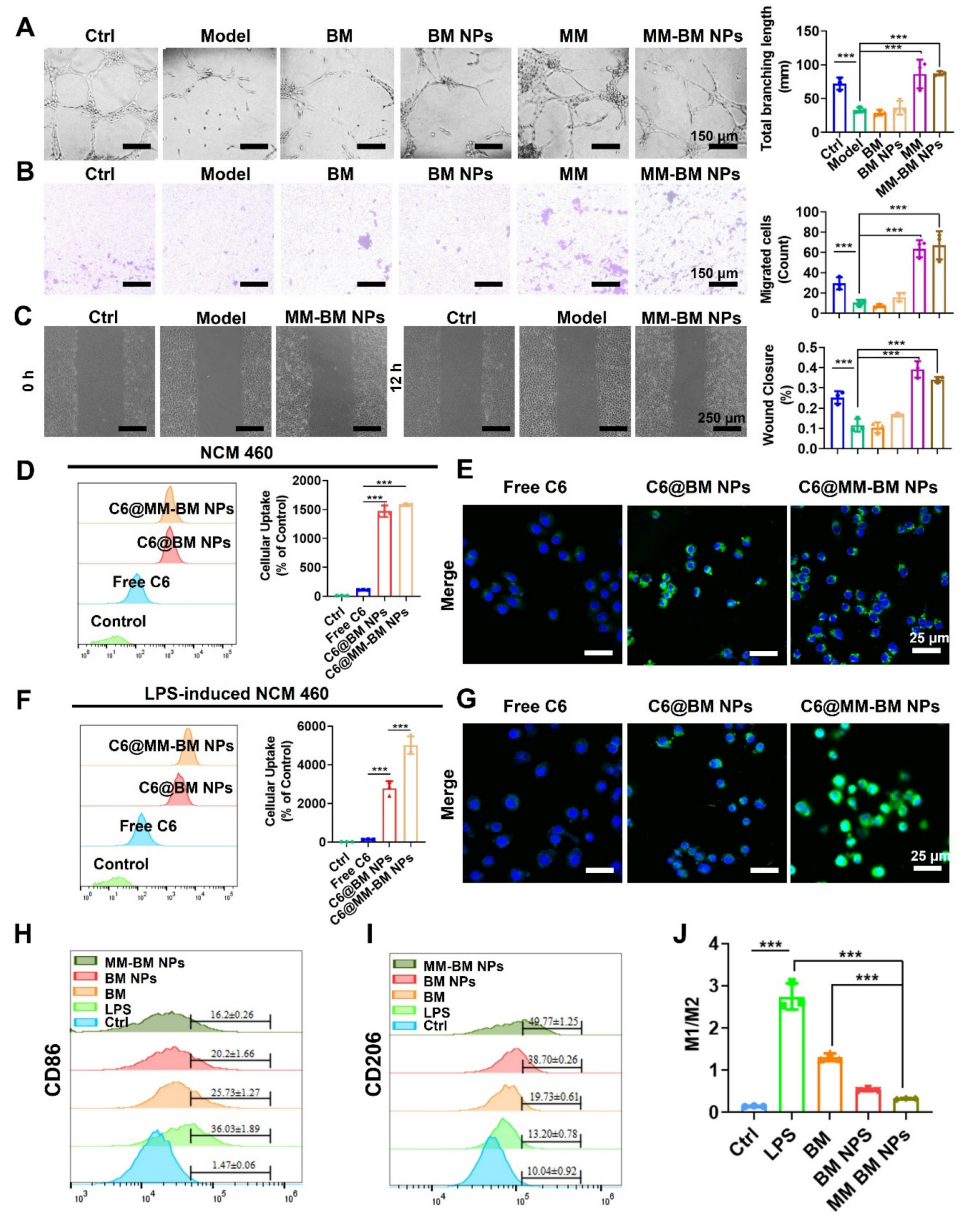
Reparative effects of MM-BM NPs on blood vessels, therapeutic effects on intestinal epithelial cells and macrophage polarization and anti-inflammatory effects *in vitro*. (A) Images and histogram of blood vessel formation experiments incubation with different preparations. (B) Images and histogram of transwell migration assays incubated with different preparations. (C) Scratch assay and histogram incubation with different preparations. (D) Flow cytometry histogram and CLSM images (E) of NCM 460 cells uptake of different formulations. (F) Flow cytometry histogram and CLSM images (G) of LPS-induced NCM 460 cells cellular uptake of different formulations. (H) Histogram of CD86 expression by different NPs. (I) Histogram of CD206 expression by different NPs. (J) Histogram of M1/M2 ratio expression by different NPs. Data are mean ± SD. *p < 0.05, **p < 0.01, ***p < 0.001.

UC is often accompanied by bleeding due to rupture of blood vessels in the colonic tissue, which exacerbates inflammation 36. Given macrophages' known capacity for vascular repair through direct adhesion and mechanical traction 37. We therefore investigated the vascular repair function of MM-BM NPs. Using tubule formation experiments, we verified that the MM group and the MM-BM NPs group could directly pro-angiogenic capacity (Figure 2A). The migration assay (Figure 2B) demonstrated a significant increase in the migration capacity of HUVECs cells, suggesting that MM can repair blood vessels by promoting traction. Compared with other groups, the MM and MM-BM NPs groups had more pronounced effects, and the total branch length was similar in the MM and MM-BM NPs groups. In addition, scratch test assays further confirmed accelerated endothelial cell migration in MM-containing treatment groups (Figures 2C and S3). Collectively, these findings demonstrate MM-BM NPs possess robust vascular repair capabilities through multiple mechanisms.

### Intestinal epithelial barrier restoration of MM-BM NPs

Repair of the IEB promotes proliferation and differentiation of intestinal stem cells, thereby impeding UC development 38, 39. To achieve targeted IEB restoration, MM-BM NPs exploit the intrinsic targeting capability of MM integrins to mediate specific adhesion to activated vascular endothelium at inflammatory sites 40. Such mechanism is also exploited by MM coated NPs to achieve active targeting of inflammation 41. Here, colon endothelial cells NCM 460 cells and LPS-induced NCM 460 cells were employed to investigate cellular uptake behavior of MM-BM NPs. As can be seen from the CLSM images shown in Figure 2D-E, the fluorescence intensity of the C6@MM-BM NPs and C6@BM NPs groups was not significantly different but higher than that of the free C6 group after treating the NCM 460 cells with different agents for 4 h. However, in LPS-induced NCM 460 cells, C6@MM-BM NPs group showed significantly higher fluorescence intensity than that of free C6 and C6@BM NPs groups (Figure 2F-G). FCM analysis further confirmed that C6@MM-BM NPs hold more cellular uptake to the inflamed NCM 460 cells than other groups. These results collectively demonstrated that the membrane proteins Integrin α4 and CD11b on MM-BM NPs can significantly promote the uptake of inflammatory cells. Meanwhile, we investigated the therapeutic effects of MM-BM NPs on inflamed NCM 460 cells. First, CCK-8 assay was conducted to evaluate the cytotoxicity of MM-BM NPs against NCM 460 cells. Compared with BM, BM NPs, and MM-BM NPs had less inhibitory effect on the proliferation of NCM 460 cells, BM NPs, and MM-BM NPs had no significant effect on the survival of NCM 460 cells at concentrations of 5-20 μM (Figure S4). BM NPs and MM-BM NPs significantly reduced the levels of TNF-α and IL-6 in NCM 460 cells, and increased the levels of IL-10 (Figure S5). The results suggest that the anti-inflammatory effects of Ber and Mag, as well as the uptake of pro-inflammatory cytokines by MM, give MM-BM NPs a synergistic anti-inflammatory effect, resulting in rapid repair of intestinal epithelial tissue.

### *In vitro* inflammatory microenvironment regulation of MM-BM NPs

The inflammatory microenvironment in UC is critically regulated by macrophage polarization and associated cytokine production. M1 macrophages produce pro-inflammatory cytokines and ROS, whereas M2 macrophages secrete anti-inflammatory cytokines, and thus M2 macrophages have been used to treat UC 42. As BM NPs can drive macrophage polarization toward M2, and MM can capture inflammatory cytokines for in situ immunity. We next investigated the ability of MM-BM NPs to regulate the inflammatory microenvironment. RAW 264.7 cells were treated with LPS to induce M1 polarization. The M1 macrophages treated with BM, BM NPs, and MM-BM NPs decreased the expression of M1 phenotype (CD86), and MM-BM NPs showed better inhibitory effects than other groups (Figure 2H). Meanwhile, MM-BM NPs also showed improved M2-polarizing effects than other formulations, as the increased level of M2 phenotype (CD206) was observed (Figure 2I). Figure 2J showed the M1/M2 ratio of macrophages, Compared with LPS group, M1/M2 ratio decreased in other treatment groups, and MM-BM NPs group was more significant. It can be proved that MM-BM NPs can effectively reprogram the polarization of RAW 264.7 cells from pro-inflammatory M1 to anti-inflammatory M2.

Relevant studies have shown that the inflammation caused by UC mainly inflammatory cytokines were secreted by macrophages 43. The CCK-8 assay revealed that the concentration of BM NPs and MM-BM NPs 1-10 μM had no significant effect on the cell viability of RAW 264.7 cells (Figure S6). We subsequently evaluated the anti-inflammatory efficacy of MM-BM NPs by quantifying key cytokines: pro-inflammatory TNF-α and IL-6, and anti-inflammatory IL-10. BM NPs and MM-BM NPs significantly reduced the levels of TNF-α and IL-6 in RAW 264.7 cells, and increased the levels of IL-10 (Figure S7). Importantly, with the increase of concentration, the therapeutic effect of MM-BM NPs was more significant than that of BM NPs, attributing to the capacity of MM to absorb inflammatory cytokine, which was previously reported. The results showed that MM-BM NPs could improve the anti-inflammatory effect of intestinal cells *in vitro*, because of both the anti-inflammatory effects of Ber and Mag and the pro-inflammatory cytokine absorbing effects of MM, which exert synergistic anti-inflammatory effects with MM-BM NPs.

### *In vivo* targeted delivery, retention and permeation of MM-BM NPs

**Figure 3:**
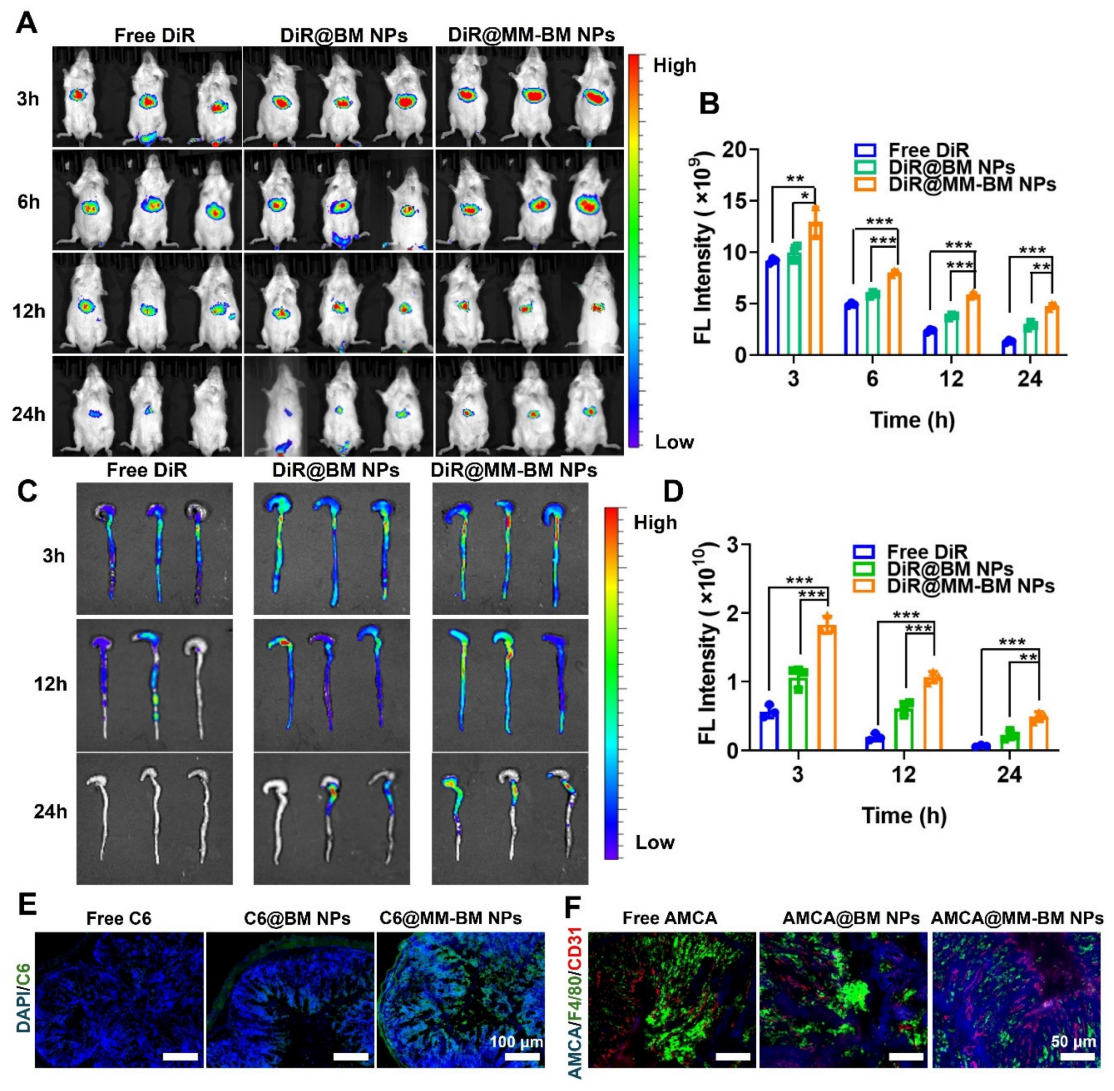
*In vivo* colon distribution and colonic tissue permeation of MM-BM NPs by intravenous administration. (A) Fluorescence images of UC mice after intravenous administration of different DiR formulations at 3, 6, 12, and 24 h. (B) Histogram of the fluorescent signal analysis of different groups. (C) Fluorescence images of colon tissues after intravenous administration of different formulations at 3, 12, and 24 h. (D) Histogram of the fluorescent signal analysis of colon tissues of different groups. (E) Fluorescence observation of colon tissue sections to investigate the tissue penetration of MM-BM NPs. (F) Fluorescence observation of colon tissue sections to investigate the distribution of MM-BM NPs on macrophages (F4/80 staining) and blood vessels (CD31 staining). Data are mean ± SD. *p < 0.05, **p < 0.01, ***p < 0.001.

To evaluate the targeting effect of BM NPs and MM-BM NPs in UC mice *in vivo*, DiR loaded NPs were intravenously injected into UC mice and monitored by IVIS (PerkinElmer) system at scheduled time point. As shown in the IVIS images and quantitative analysis (Figure 3A-B), the fluorescence intensity of the DiR@MM-BM NPs group in the colon was significantly higher than that of the other groups at 3 h post-injection. At each test time point, the signal intensity of DiR@MM-BM in the colon site was higher than that of other groups. The results showed that DiR@MM-BM NPs exhibited much better accumulation in the colon than free DiR and DiR@BM NPs. Meanwhile, the colon and main organs were collected for *in vivo* imaging at scheduled time point (Figure 3C-D, and S8). The strongest fluorescence intensity was observed in the colon of the DiR@MM-BM NPs group compared to the other groups, which was attributed to the homing effect of macrophage-mimicking NPs and reduced reticuloendothelial system (RES) clearance 44. The results showed that DiR@MM-BM NPs had enhanced inflammatory colonic targeting efficiency by MM camouflage strategy.

To further validate the inflammatory targeting advantages of MM-BM NPs, we further compared the distribution profiles of DiR@MM-BM NPs and other counterparts in normal mice. As shown in Figure S9, after we injected different DiR formulations into normal mice via tail vein, only a small amount of either DiR@BM NPs or DiR@MM-BM NPs could accumulate in colon tissues. It was greatly unlike the distribution profiles in colitis mice, due to the absence of GVB disruption in normal mice. Additionally, since there was no inflammatory lesion in colon tissue, DiR@MM-BM NPs did not exhibit the inflammatory targeting distribution, compared to DiR@BM NPs.Therefore, this result further demonstrated the specific inflammatory targeting of MM-BM NPs.

To further investigate the retention and permeation of MM-BM NPs in the colon, the colon was taken 12 hours after administration of free C6, C6@BM NPs, and C6@MM-BM NPs to UC mice, respectively, and the colon was evaluated by CLSM after frozen sectioning. As shown in Figure 3E, the green fluorescence signals in the MM-BM NPs treated colon were stronger than those in the BM NPs group, suggesting that the MM camouflage strategy could help BM NPs penetrate and absorb into the colonic tissues efficiently, suggesting that MM-BM NPs were highly selective for the colonic inflammation site. These findings suggested a mechanism that MM-BM NPs had MM functional proteins that can actively target inflammatory cells, thereby achieving drug accumulation and absorption 45.

As shown in Figure 3F, blue fluorescence derived from AMCA@MM-BM NPs could well overlap with the green fluorescence and red fluorescence, indicated macrophages and blood vessels respectively. It reflected that MM coating promoted BM NPs to target the site of vascular breakage and well retained at the blood vessels. MM coating has the direct adhesion effect on blood vessels. It also can recruit more macrophages to reach the site of vascular injury and accelerate the repair of blood vessels. Overall, the results suggested that MM-BM NPs can ultimately promote GVB repairing mediated by direct adhesion and mechanical traction.

### Therapeutic efficacy of MM-BM NPs in DSS-induced UC mice

**Figure 4:**
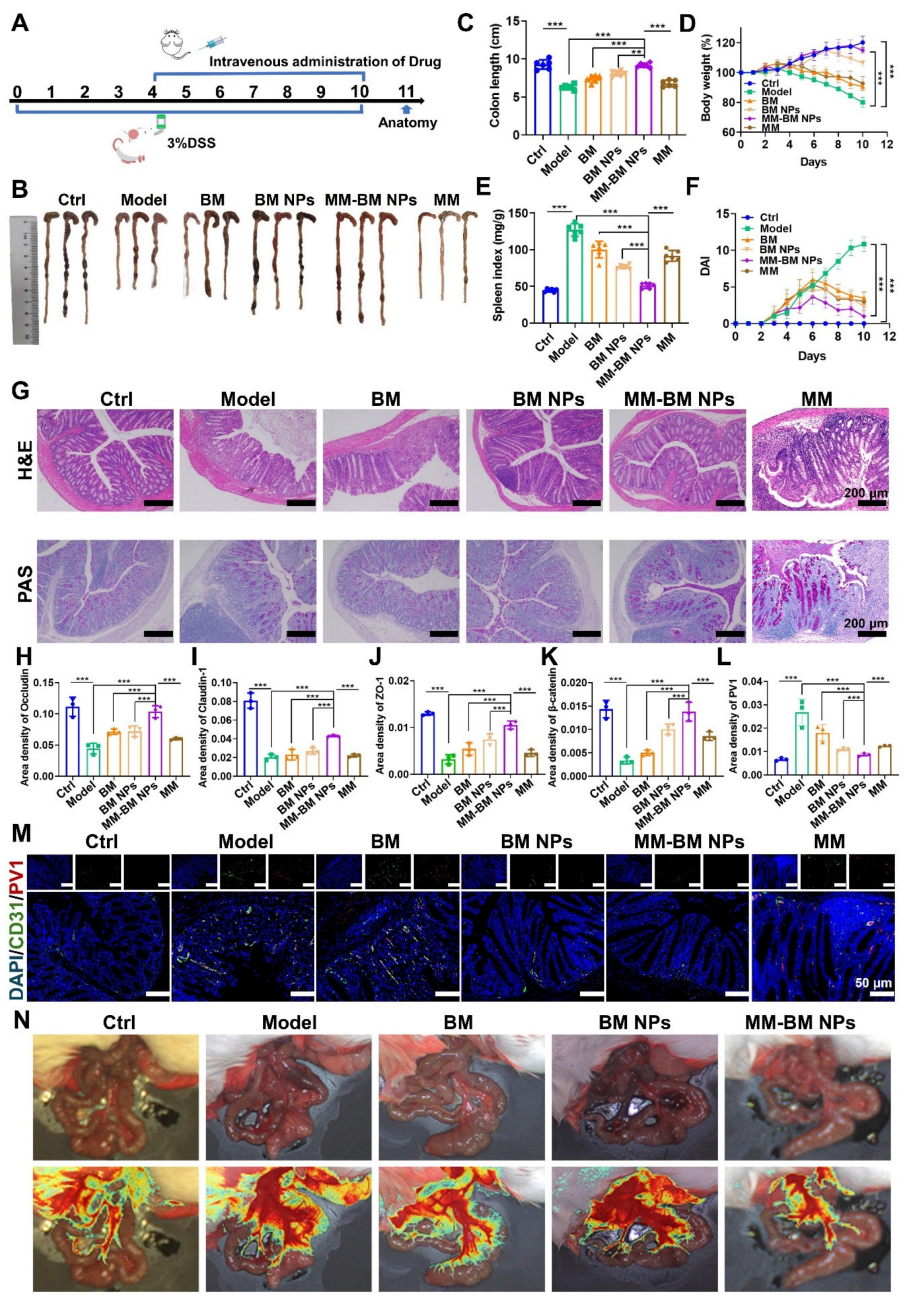
MM-BM NPs significantly attenuated DSS-induced colitis and double-bowel barrier repair. (A) Experimental scheme. (B) Representative photographs of colon tissues. (C) Histogram assessment of colon length. (D) Body weight of mice. (E) Histogram evaluation of spleen index. (F) Graph of DAI scores. (G) H&E and PAS staining of colonic sections. Quantitative analysis of immunohistochemistry Occludin (H), Claudin-1 (I), ZO-1 (J), β-catenin (K), and PV1 (L). (M) Immunofluorescent analysis of DAPI, CD31, and PV1 in each group. (N) Visualization of microcirculation by laser Doppler perfusion imaging. Data are mean ± SD. *p < 0.05, **p < 0.01, ***p < 0.001.

A mice UC model was established by DSS to evaluate the therapeutic effect of MM-BM NPs *in vivo*. The experimental protocol was illustrated in Figure 4A. Shortened colon length was a typical symptom of DSS-induced UC 46. As shown in Figure 4B, C markedly shortened the colon length was observed in DSS induce mice group, while MM-BM NPs distinctly recovered the colon length, similar to that of normal mice. As shown in Figure 4D, intravenous administration of BM NPs and MM-BM NPs effectively prevented weight loss, among which MM-BM NPs showed a better effect. Moreover, the spleen index also reflected UC inflammation 47, 48. The spleen index of mice in the DSS induced Model group was significantly increased, indicating severe inflammatory symptoms. All agents showed varying degrees of splenic index relief, with MM-BM-NPs being the most potent (Figure 4E). Compared to other counterparts, MM-BM NPs treatment also significantly decreased DAI (Figure 4F). Interesting, we added the MM alone treatment in this experiment to evaluate the therapeutic efficacy of MM group. To a certain extent, alone MM treatment could restore the length of the colon, ameliorate weight loss, reduce DAI, and lower the spleen index. It indicated that MM can influence gut barriers independently of BM NPs. In conclusion, the therapeutic effects of MM-BM NPs on colonic tissue-related indicators were all different from those of other groups, indicating that MM-BM NPs had a significant therapeutic effect on DSS induced UC mice.

Histological analysis such as H&E and PAS staining can identify inflammatory damage to colon tissue 49, 50. As shown in Figure 4G, colon tissue in Model group showed significant edema, serious loss of mucosal epithelium, extensive infiltration of central granulocytes, and necrosis of intestinal crypts disappeared. Compared with the Model group, BM NPs and MM-BM NPs all showed alleviated inflammatory severities, and the therapeutic effect of MM-BM NPs showed the best histological conditions in the colon. Among these, the MM group also demonstrated a certain degree of reduction in inflammation. At the same time, tissue slices of the major organs (heart, liver, spleen, lungs, and kidneys) in the BM, BM NPs, and MM-BM NPs groups showed no significant pathological abnormalities or injuries (Figure S10), indicating that MM-BM NPs had good biocompatibility and low toxicity.

The IEB plays a pivotal role in modulating UC-associated inflammation, with tight junction proteins (Occludin, Claudin-1, and ZO-1) serving as key molecular indicators of barrier integrity 51, 52. Immunohistochemical results showed that the expressions of Occludin, Claudin-1, and ZO-1 were significantly reduced in the Model group compared to the control group (Figure 4H-J, and S11). MM-BM NPs significantly increased the expression of ZO-1, Occludin, and Claudin-1, which were significantly better than BM and BM NPs. Among these, the MM group provided some degree of protection for intestinal tight junction complexes. These findings demonstrate that MM-BM NPs provide exceptional protection of intestinal tight junction complexes, thereby facilitating IEB restoration.

Plasmalemma vesicle-associated protein 1 (PV1) is a vascular endothelium-specific transmembrane protein associated with transmembrane crossing between endothelial cells, and its expression level correlates with disruption of the integrity of the GVB. β-catenin signaling activation attenuates PV1 expression on CD31 endothelium, which serves as a critical mechanism for maintaining GVB homeostasis 10. Notably, the Model group showed a significant increase in PV1 expression and a significant decrease in β-catenin, whereas MM-BM NPs effectively reversed the increase in PV1 and decrease in β-catenin (Figure 4K-L, and S12). Among these, the MM group effectively suppressed PV1 expression and promoted β-catenin expression. Immunofluorescence analysis further revealed that MM-BM NPs administration significantly reduced both CD31 and PV1 expression in colonic tissues compared to controls (Figure 4M), indicating suppression of pathological angiogenesis. Among these, the MM group effectively reduced the expression of CD31 and PV1 in colonic tissue. These findings collectively demonstrate that MM-BM NPs promotes GVB repair through a dual mechanism of reducing PV1 and β-catenin levels and inhibiting disease-related neovascularization.

Laser speckle perfusion imaging was utilized to confirm the establishment of double intestinal barrier disruption caused by UC. The results showed severe blood leakage in the colon of the Model group. Improvement in mucosal microcirculatory blood flow after treatment with MM-BM NPs was assessed. In addition, the intestinal tissues in the Model group also showed congestion, exudation, and edema, which were alleviated after treatment with MM-BM NPs (Figure 4N). Collectively, these findings demonstrate that MM-BM NPs provide comprehensive barrier restoration, effectively repairing both the IEB and GVB through coordinated mechanisms.

### Regulation of the intestinal microbiota of MM-BM NPs

After verifying the dual intestinal barriers repair function of MM-BM NPs of UC *in vivo*, we investigated whether the intestinal flora homeostasis of UC mice was restored. The 16S rRNA gene is the most used molecular marker for systematic taxonomic studies of prokaryotic microorganisms, allowing precise quantification of all species of gut microorganisms 53. So, we investigated the regulation of the intestinal microbiota of different agents on the composition and abundance of intestinal flora using 16S RNA gene sequencing. As shown in Figure S13A, the species composition was significantly reduced in UC mice, whereas MM-BM NPs significantly restored the alpha diversity of the gut microbiota. Alpha Diversity dilution curves showed good diversity of gut flora between groups, indicating that the sequencing was deep enough to cover the sample species and avoid systematic errors (Figure S13B). The results of PCoA showed that the microbial communities in the MM-BM NPs group differed significantly from those in the Model group and were like those in the control group (Figure S13C), suggesting that MM-BM NPs had a better effect in regulating microbial communities. The MM-BM NPs group was more like the control OTUs, suggesting that the species composition of the MM-BM NPs group was closer to that of the control group (Figure S13D). The developmental tree (Figure S13E) also showed similar results, suggesting that the microbiota in the MM-BM NPs group was more like the control group. At the family level (Figure S13F), the abundance of *Lachnospiraceae* and *Muribaculaceae* in the Model group was significantly decreased compared with that in the control group, whereas *Enterobacteraceae*_A abundance increased. At the phylum level (Figure S13G), the abundance of *Bacteroidota* and *Firmicutes_*A were significantly decreased, while the abundance of *Verrucomicrobiota* was significantly increased in the Model group compared to the control group. All the administered groups improved the abnormal intestinal flora structure in UC mice. Among them, the MM-BM NPs group had better regulation, and the overall gut flora distribution was more like that of the control group. Notably, MM-BM NPs-mediated microbiota modulation correlated with improved inflammatory profiles, increased *Bacteroidota* and decreased *Proteobacteria* abundance were able to reduce inflammatory factor production. Increasing *Patescibacteria* reduced inflammatory factor production and promoted anti-inflammatory IL-10 secretion (Figure S13H). These findings suggest that MM-BM NPs can successfully reach the site of colonic inflammation and achieve therapeutic efficacy by coordinating the restoration of gut microbiota composition and barrier function.

Based on previous studies, the liver and pancreas were prioritized for detecting gut bacterial translocation due to their unique vulnerability as direct anatomical targets of gut-derived pathogens in UC. The liver, receiving ~ 80% of its blood via the portal vein, acts as the primary filter for bacteria escaping the compromised GVB. Bacterial accumulation here directly triggers Kupffer cell activation and systemic inflammation (TNF-α/IL-6). The pancreas, sharing developmental pathways with the gut, is highly susceptible to BT-induced injury, where translocated bacteria (e.g., *Bacteroides*) activate proteases and inflammatory cascades (MCP-1/IL-8). Detection in these organs provides clinically actionable evidence linking GVB failure to systemic inflammation. Therefore, UC-induced disruption of the intestinal bi-intestinal barrier causes translocation of intestinal microorganisms, which leads to activation of hepatic inflammation via the gut-hepatic axis and pancreatic inflammation via the gut-pancreatic axis 54, 55. We further investigated the flora of the liver and pancreas after bacterial translocation caused by UC by MM-BM NPs. Subsequently, the flora composition of the liver and pancreas was analyzed at the portal level and at the genus level. There was a high similarity in the microbial composition between the control and MM-BM NPs groups based on the liver and pancreas phylum level abundance. *Proteobacteria* phylum, one of the major bacterial phyla, is associated with the onset and progression of several inflammatory conditions. *Proteobacteria* phylum causes inflammation and infection when it reaches sites other than the intestinal tract. *Bacteroidota* phylum and *Proteobacteria* phylum were significantly higher in the Model group, while MM-BM NPs treatment was effective in suppressing the abundance of *Bacteroidota* phylum and *Proteobacteria* phylum in the liver and pancreas (Figure S13I and K). Harmful bacteria *Acinetobacter* was found to be significantly elevated in the Model group based on liver genus level abundance, and *Acinetobacter* abundance was improved in all other groups (Figure S13J). The abundance of harmful bacteria *Acinetobacter*, *Bacteroides H*, and *Escherichia 710834* were found to be significantly elevated in the Model group according to the pancreas genus level abundance, in which the MM-BM NPs group was able to significantly down-regulate the abundance of the above harmful bacteria (Figure S13L). These changes in bacterial abundance in the liver and pancreas suggest that the good ability of MM-BM NPs to repair the intestinal double barrier hinders bacterial translocation to liver and pancreas infections and facilitates hepatic and pancreatic inflammation to subside. Meanwhile, it illustrates the potential of hepatic and pancreatic therapy by interfering with the intestinal flora.

### *In vivo* inflammatory microenvironment regulation of MM-BM NPs

**Figure 5:**
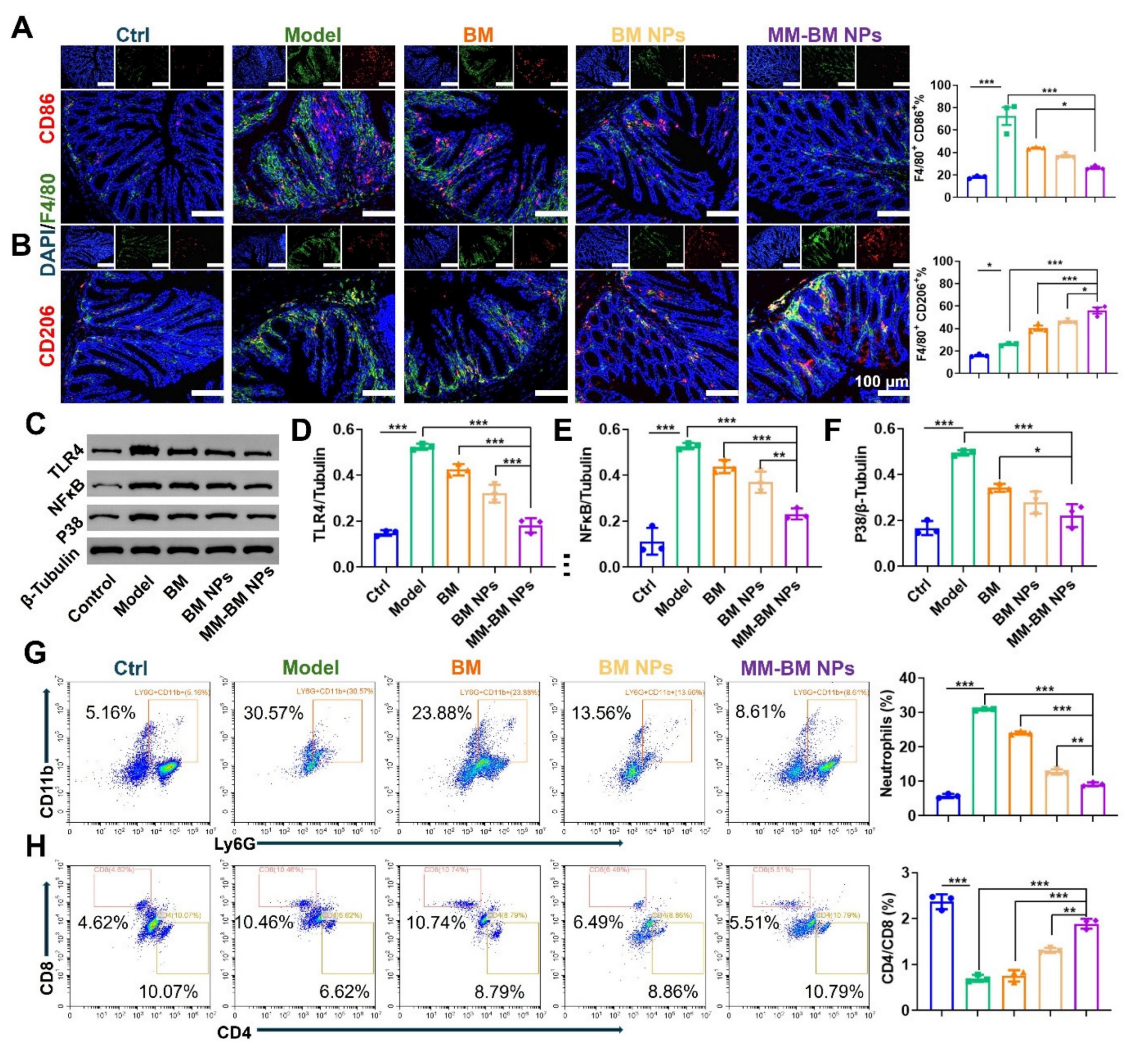
Regulation of Immune regulation by different NPs *in vivo*. (A)Immunofluorescent analysis and FCM Quantitative analysis of DAPI, F4/80, and CD86. (B) Immunofluorescent analysis and FCM Quantitative analysis of DAPI, F4/80, and CD206. (C) Western blotting results of TLR4, NF-κB, and P38 in the colon of each group. Histogram analysis of different preparations on the changes of TLR4 (D), NF-κB (E), and P38 (F) expression. (G) Flow diagram and quantification analysis of colonic neutrophils. (H) Flow diagram and quantification analysis of colonic CD4/CD8 T cells. Data are mean ± SD. *p < 0.05, **p < 0.01, ***p < 0.001.

The TLR4/NF-κB/MAPK signaling pathway represents a crucial regulatory axis in macrophage polarization and function 56. This pathway not only governs cytokine production in ulcerative colitis (UC) but also mediates intestinal inflammatory and immune responses. Mechanistically, TLR4 serves as the upstream activator of NF-κB, triggering pro-inflammatory cytokine secretion that exacerbates UC pathogenesis 57. Furthermore, NF-κB plays pivotal roles in maintaining intestinal immune homeostasis and modulating inflammatory responses, while p38 functions as the central regulator of MAPK signaling 58. To explore the therapeutic effect of MM-BM NPs on UC by modulating macrophage polarization, CD86, CD206, TLR4, NF-κB, and P38 were measured by immunofluorescence, flow cytometry, and western blotting. As shown in Figure 5(A-B, and S14), the results of immunofluorescence and flow cytometry showed that the fluorescence intensity of CD86 was inhibited by different treatments, and the MM-BM NPs inhibited CD86 more strongly. In contrast, MM-BM NPs promoted CD206 expressions most significantly. In addition, the western blot results were shown in Figure 5C-F, where the protein expressions of TLR4 and NF-κB were significantly lower in the MM-BM NPs group compared to the BM and BM NPs groups, and the protein expression of P38 was significantly lower than that in the BM group. In conclusion, MM-BM NPs could effectively regulate the polarization process of macrophages by regulating the TLR4/NF-κB/MAPK signaling pathway and ultimately achieving effective treatment of UC.

Hyperactivation of immune cells, such as neutrophils and helper T cells, is a central process in the formation and progression of UC 59-61. Thus, *in vivo* regulation of these immune cells by MM-BM NPs was investigated. As shown in Figure 5G, the proportion of neutrophils in CD45 cells of UC mice was significantly higher than that in the control group, suggestive inflammatory microenvironment in UC, which was suppressed by MM-BM NPs, with decreasing of nearly 4-fold and 3-fold, compared with the Model group and BM group, respectively. Meanwhile, the addition of MM-BM NPs significantly increased the proportion of the CD4/CD8 in T cells (1.89%), in comparison to the BM (0.75%), BM NPs (1.30%) and Model group (0.70%) (Figure 5H).

Amount of inflammatory cytokines determines UC severity 62. The severity of UC was directly correlated with the number of inflammatory cytokines. The pro-inflammatory cytokines (TNF-α and IL-6) were significantly higher in the colonic tissues of mice in the DSS-induced group than those in the control group (Figure S15). MM-BM NPs significantly reduced the levels of TNF-α and IL-6, and the inhibitory effect was superior to that of BM NPs and BM treatment groups. The anti-inflammatory cytokine IL-10 showed an opposite trend to TNF-α and IL-6. After treatment with MM-BM NPs, IL-10 levels were significantly higher than in BM NPs and BM treatment groups (Figure S15). These data again demonstrate the immunotherapeutic potential of MM-BM NPs, which is consistent with the *in vitro* anti-inflammatory results.

## Conclusion

A promising dual-barrier therapeutic platform is currently needed for the treatment of UC. In this study, we successfully developed a multifunctional biomimetic drug self-assembly platform of MM for UC, aiming at rapid repair of GVB and IEB, and better restoration of the intestinal immune microenvironment and intestinal flora disorders. Considering that oral drug delivery systems are susceptible to degradation in the gastrointestinal tract and mostly only effectively improve the IEB while struggling to repair the colonic GVB, this study employed MM membrane-coated BM NPs administered intravenously. Leveraging the inflammatory targeting mediation of the MM membrane, the MM-BM NPs efficiently accumulated in the damaged colonic vasculature and penetrated the colon tissue, achieving dual repair of both the GVB and IEB. *In vitro*, MM promotes the tube-forming and migratory abilities of HUVECs cells, while MM neutralizes inflammatory cytokines and has specific proteins on its surface that bind to inflammatory cells. And MM-BM NPs inherited the ability of MM and synergistically led to polarization of M1 macrophages like M2 macrophages to regulate the inflammatory. *In vivo* biodistribution showed that the MM coating in MM-BM NPs improved the targeting and retention capacity of inflamed intestinal epithelial and intestinal vascular tissues due to the MM coating in MM-BM NPs. Thus, MM guided the BM NPs to the inflamed colonic tissues and the damaged vascular tissues to perform vascular repair on one hand, and to neutralize a variety of pro-inflammatory factors on the other hand. The released BM NPs exerted intestinal epithelial repair, anti-inflammatory effects and regulation of flora balance. This modality significantly improved the restoration of the dual barriers and had a good therapeutic effect on DSS-induced UC mice by decreasing the degree of inflammation, regulating macrophage polarization, modulating immune cells and improving intestinal flora. Overall, the synergistic restoration of the intestinal dual barriers, modulation of the inflammatory microenvironment, and restoration of the intestinal flora homeostasis were achieved by the biomimetic self-assembly nano-strategy, which provides a new prospect for the treatment of UC.

## Supplementary Material

Supplementary figures.

## References

[1] Ungaro R, Mehandru S, Allen PB, Peyrin-Biroulet L, Colombel JF (2017). Ulcerative colitis. Lancet.

[2] Kelly CR, Ananthakrishnan AN (2019). Manipulating the Microbiome With Fecal Transplantation to Treat Ulcerative Colitis. JAMA.

[3] Tatiya-Aphiradee N, Chatuphonprasert W, Jarukamjorn K (2018). Immune response and inflammatory pathway of ulcerative colitis. J Basic Clin Physiol Pharmacol.

[4] Liu ZH, Liu H, Cheng JL, Wang HL, Yang YF, Le J (2024). Strategies and opportunities of micro/nano delivery systems for targeted therapy of ulcerative colitis: Focus on underlying mechanisms and future perspectives. Chin Chem Lett.

[5] Eftychi C, Schwarzer R, Vlantis K, Wachsmuth L, Basic M, Wagle P (2019). Temporally Distinct Functions of the Cytokines IL-12 and IL-23 Drive Chronic Colon Inflammation in Response to Intestinal Barrier Impairment. Immunity.

[6] Liu FJ, Meng FS, Yang ZJ, Wang H, Ren YH, Cai Y (2024). Exosome-biomimetic nanocarriers for oral drug delivery. Chin Chem Lett.

[7] Jin T, Lu HY, Zhou Q, Chen DF, Zeng YY, Shi JY (2024). HS-Releasing Versatile Montmorillonite Nanoformulation Trilogically Renovates the Gut Microenvironment for Inflammatory Bowel Disease Modulation. Adv Sci (Weinh).

[8] Pabst O, Hornef MW, Schaap FG, Cerovic V, Clavel T, Bruns T (2023). Gut-liver axis: barriers and functional circuits. Nat Rev Gastroenterol Hepatol.

[9] Thomas H (2016). Gut microbiota: Microbiota promote gut healing. Nat Rev Gastroenterol Hepatol.

[10] Spadoni I, Zagato E, Bertocchi A, Paolinelli R, Hot E, Di Sabatino A (2015). A gut-vascular barrier controls the systemic dissemination of bacteria. Science.

[11] Bertocchi A, Carloni S, Ravenda PS, Bertalot G, Spadoni I, Lo Cascio A (2021). Gut vascular barrier impairment leads to intestinal bacteria dissemination and colorectal cancer metastasis to liver. Cancer Cell.

[12] Carloni S, Bertocchi A, Mancinelli S, Bellini M, Erreni M, Borreca A (2021). Identification of a choroid plexus vascular barrier closing during intestinal inflammation. Science.

[13] Bao M, Wang K, Li J, Li Y, Zhu H, Lu M (2023). ROS Scavenging and inflammation-directed polydopamine nanoparticles regulate gut immunity and flora therapy in inflammatory bowel disease. Acta Biomater.

[14] Han X, Luo R, Qi S, Wang Y, Dai L, Nie W (2023). "Dual sensitive supramolecular curcumin nanoparticles" in "advanced yeast particles" mediate macrophage reprogramming, ROS scavenging and inflammation resolution for ulcerative colitis treatment. J Nanobiotechnology.

[15] Li D, Feng Y, Tian M, Ji J, Hu X, Chen F (2021). Gut microbiota-derived inosine from dietary barley leaf supplementation attenuates colitis through PPARγ signaling activation. Microbiome.

[16] Langer V, Vivi E, Regensburger D, Winkler TH, Waldner MJ, Rath T (2019). IFN-γ drives inflammatory bowel disease pathogenesis through VE-cadherin-directed vascular barrier disruption. J Clin Invest.

[17] Cheng W, Wang X, Wu Y, Li W, Fu C, Zou L (2023). Huanglian-Houpo extract attenuates DSS-induced UC mice by protecting intestinal mucosal barrier and regulating macrophage polarization. J Ethnopharmacol.

[18] Park J, Wu Y, Le QV, Kim JS, Xu E, Lee J (2025). Self-disassembling nanoparticles as oral nanotherapeutics targeting intestinal microenvironment. Nat Commun.

[19] Huang L, Hu W, Huang LQ, Zhou QX, Song ZY, Tao HY (2024). "Two-birds-one-stone" oral nanotherapeutic designed to target intestinal integrins and regulate redox homeostasis for UC treatment. Sci Adv.

[20] Fan X, Zhang ZZ, Gao WX, Pan QQ, Luo K, He B (2023). An Engineered Butyrate-Derived Polymer Nanoplatform as a Mucosa-Healing Enhancer Potentiates the Therapeutic Effect of Magnolol in Inflammatory Bowel Disease. ACS Nano.

[21] Wang X, Jiao M, Tian F, Lu X, Xiong H, Liu F (2023). A Biomimetic Nanoplatform with Improved Inflammatory Targeting Behavior for ROS Scavenging-Based Treatment of Ulcerative Colitis. Adv Healthc Mater.

[22] Amirthalingam S, Kim S, Roo D, Ryu KM, Jeong J, Hennebert PM (2024). Biomimetic nanocomposite cryogel with enhanced peptide binding promotes therapeutic angiogenesis and bone regeneration. Chem Eng J.

[23] Chen S, Saeed A, Liu Q, Jiang Q, Xu H, Xiao GG (2023). Macrophages in immunoregulation and therapeutics. Signal Transduct Target Ther.

[24] Huang X, Wang LT, Guo HY, Zhang WY (2023). Macrophage membrane-coated nanovesicles for dual-targeted drug delivery to inhibit tumor and induce macrophage polarization. Bioact Mater.

[25] Zhang Q, Zhou J, Zhou J, Fang RH, Gao W, Zhang L (2021). Lure-and-kill macrophage nanoparticles alleviate the severity of experimental acute pancreatitis. Nat Commun.

[26] Shan B, Zhou Y, Yin M, Deng Y, Ge C, Liu Z (2023). Macrophage Membrane-Reversibly Cloaked Nanotherapeutics for the Anti-Inflammatory and Antioxidant Treatment of Rheumatoid Arthritis. Small Methods.

[27] Xiao TT, He MJ, Xu F, Fan Y, Jia BY, Shen MW (2021). Macrophage Membrane-Camouflaged Responsive Polymer Nanogels Enable Magnetic Resonance Imaging-Guided Chemotherapy/Chemodynamic Therapy of Orthotopic Glioma. ACS Nano.

[28] Li RX, He YW, Zhu Y, Jiang LX, Zhang SY, Qin J (2019). Route to Rheumatoid Arthritis by Macrophage-Derived Microvesicle-Coated Nanoparticles. Nano Lett.

[29] Liu C, Wu C, Yang Q, Gao J, Li L, Yang D (2016). Macrophages Mediate the Repair of Brain Vascular Rupture through Direct Physical Adhesion and Mechanical Traction. Immunity.

[30] Fantin A, Vieira JM, Gestri G, Denti L, Schwarz Q, Prykhozhij S (2010). Tissue macrophages act as cellular chaperones for vascular anastomosis downstream of VEGF-mediated endothelial tip cell induction. Blood.

[31] Yu Y, Dai K, Gao Z, Tang W, Shen T, Yuan Y (2021). Sulfated polysaccharide directs therapeutic angiogenesis via endogenous VEGF secretion of macrophages. Sci Adv.

[32] Zeng R, Lv B, Lin Z, Chu X, Xiong Y, Knoedler S (2024). Neddylation suppression by a macrophage membrane-coated nanoparticle promotes dual immunomodulatory repair of diabetic wounds. Bioact Mater.

[33] Gao C, Huang Q, Liu C, Kwong CHT, Yue L, Wan JB (2020). Treatment of atherosclerosis by macrophage-biomimetic nanoparticles via targeted pharmacotherapy and sequestration of proinflammatory cytokines. Nat Commun.

[34] Thamphiwatana S, Angsantikul P, Escajadillo T, Zhang Q, Olson J, Luk BT (2017). Macrophage-like nanoparticles concurrently absorbing endotoxins and proinflammatory cytokines for sepsis management. Proc Natl Acad Sci U S A.

[35] Wang H, Liu H, Li J, Liu C, Chen H, Li J (2023). Cytokine nanosponges suppressing overactive macrophages and dampening systematic cytokine storm for the treatment of hemophagocytic lymphohistiocytosis. Bioact Mater.

[36] Matsumoto K, Yamaba R, Inoue K, Utsumi D, Tsukahara T, Amagase K (2018). Transient receptor potential vanilloid 4 channel regulates vascular endothelial permeability during colonic inflammation in dextran sulphate sodium-induced murine colitis. Br J Pharmacol.

[37] Ferraro B, Leoni G, Hinkel R, Ormanns S, Paulin N, Ortega-Gomez A (2019). Pro-Angiogenic Macrophage Phenotype to Promote Myocardial Repair. J Am Coll Cardiol.

[38] Pan HX, Ding BY, Jiang ZJ, Wang J, Li DW, Yu FN (2024). Hydrogel Derived from Decellularized Porcine Small Intestinal Submucosa as a Physical Shielding Repaired the Gut Epithelial Barrier of Murine Ulcerative Colitis. Adv Funct Mater.

[39] Li W, Chen D, Zhu Y, Ye Q, Hua Y, Jiang P (2024). Alleviating Pyroptosis of Intestinal Epithelial Cells to Restore Mucosal Integrity in Ulcerative Colitis by Targeting Delivery of 4-Octyl-Itaconate. ACS Nano.

[40] Lin QY, Bai J, Zhang YL, Li HH (2023). Integrin CD11b Contributes to Hypertension and Vascular Dysfunction Through Mediating Macrophage Adhesion and Migration. Hypertension.

[41] Wu Y, Wan S, Yang S, Hu H, Zhang C, Lai J (2022). Macrophage cell membrane-based nanoparticles: a new promising biomimetic platform for targeted delivery and treatment. J Nanobiotechnology.

[42] Pi Y, Wu Y, Zhang X, Lu D, Han D, Zhao J (2023). Gut microbiota-derived ursodeoxycholic acid alleviates low birth weight-induced colonic inflammation by enhancing M2 macrophage polarization. Microbiome.

[43] Shin AE, Tesfagiorgis Y, Larsen F, Derouet M, Zeng PYF, Good HJ (2023). F4/80(+)Ly6C(high) Macrophages Lead to Cell Plasticity and Cancer Initiation in Colitis. Gastroenterology.

[44] Wang D, Jiang S, Zhang F, Ma S, Heng BC, Wang Y (2021). Cell Membrane Vesicles with Enriched CXCR4 Display Enhances Their Targeted Delivery as Drug Carriers to Inflammatory Sites. Adv Sci (Weinh).

[45] Tang TT, Lv LL, Wang B, Cao JY, Feng Y, Li ZL (2019). Employing Macrophage-Derived Microvesicle for Kidney-Targeted Delivery of Dexamethasone: An Efficient Therapeutic Strategy against Renal Inflammation and Fibrosis. Theranostics.

[46] Sinha A, Li Y, Mirzaei MK, Shamash M, Samadfam R, King IL (2022). Transplantation of bacteriophages from ulcerative colitis patients shifts the gut bacteriome and exacerbates the severity of DSS colitis. Microbiome.

[47] Wang X, Gu H, Zhang H, Xian J, Li JJ, Fu CM (2021). Oral Core-Shell Nanoparticles Embedded in Hydrogel Microspheres for the Efficient Site-Specific Delivery of Magnolol and Enhanced Antiulcerative Colitis Therapy. ACS Appl Mater Interfaces.

[48] Zhang C, Li QR, Xing JH, Yang Y, Zhu MM, Lin LT (2024). Tannic acid and zinc ion coordination of nanase for the treatment of inflammatory bowel disease by promoting mucosal repair and removing reactive oxygen and nitrogen species. Acta Biomater.

[49] Zhang C, Li JX, Xiao M, Wang D, Qu Y, Zou L (2022). Oral colon-targeted mucoadhesive micelles with enzyme-responsive controlled release of curcumin for ulcerative colitis therapy. Chin Chem Lett.

[50] Li QR, Zhang C, Zhu MM, Shan J, Qian HS, Ma Y (2024). W-GA nanodots restore intestinal barrier functions by regulating flora disturbance and relieving excessive oxidative stress to alleviate colitis. Acta Biomater.

[51] Kotla NG, Isa ILM, Rasala S, Demir S, Singh R, Baby BV (2022). Modulation of Gut Barrier Functions in Ulcerative Colitis by Hyaluronic Acid System. Adv Sci (Weinh).

[52] Yan Y, Song JW, Liu DD, Liu ZH, Cheng JL, Chen ZY (2024). Simple and versatile in situ thermo-sensitive hydrogel for rectal administration of SZ-A to alleviate inflammation and repair mucosal barrier in ulcerative colitis. Chin Chem Lett.

[53] Tong L, Hao H, Zhang Z, Lv Y, Liang X, Liu Q (2021). Milk-derived extracellular vesicles alleviate ulcerative colitis by regulating the gut immunity and reshaping the gut microbiota. Theranostics.

[54] Tilg H, Adolph TE, Trauner M (2022). Gut-liver axis: Pathophysiological concepts and clinical implications. Cell Metab.

[55] Lei Y, Tang L, Liu S, Hu S, Wu L, Liu Y (2021). Parabacteroides produces acetate to alleviate heparanase-exacerbated acute pancreatitis through reducing neutrophil infiltration. Microbiome.

[56] Zhu B, Gu G, Ren J, Song X, Li J, Wang C (2023). Schwann Cell-Derived Exosomes and Methylprednisolone Composite Patch for Spinal Cord Injury Repair. ACS Nano.

[57] Mukherjee T, Kumar N, Chawla M, Philpott DJ, Basak S (2024). The NF-κB signaling system in the immunopathogenesis of inflammatory bowel disease. Sci Signal.

[58] Nyati KK, Masuda K, Mahabub-Uz Zaman M, Dubey PK, Millrine D, Chalise JP (2017). TLR4-induced NF-κB and MAPK signaling regulate the IL-6 mRNA stabilizing protein Arid5a. Nucleic Acids Res.

[59] Geremia A, Biancheri P, Allan P, Corazza GR, Di Sabatino A (2014). Innate and adaptive immunity in inflammatory bowel disease. Autoimmun Rev.

[60] Gao S, Zheng HC, Xu SJ, Kong JW, Gao F, Wang ZJ (2023). Novel Natural Carrier-Free Self-Assembled Nanoparticles for Treatment of Ulcerative Colitis by Balancing Immune Microenvironment and Intestinal Barrier. Adv Healthc Mater.

[61] Zhang N, Sun QQ, Li JH, Li J, Tang L, Zhao Q (2024). A lipid/PLGA nanocomplex to reshape tumor immune microenvironment for colon cancer therapy. Regen Biomater.

[62] Zhang C, Wang X, Xiao M, Ma JQ, Qu Y, Zou L (2022). Nano-in-micro alginate/chitosan hydrogel via electrospray technology for orally curcumin delivery to effectively alleviate ulcerative colitis. Mater Des.

