# Supplementary material for: Multifunctional macrophage mimetic nanoplatform modulates vascular and epithelial double gut barriers to alleviate ulcerative colitis
Source: Theranostics. 2026 Jan 1;16(1):58–76. doi: 10.7150/thno.118236 (PMC12665101; doi:10.7150/thno.118236)
Supplement: Supplementary file 1 — Supplementary figures. [file thnov16p0058s1.pdf]

Macrophage Membrane-Camouflaged Nanotherapy for Dual-Barrier Restoration in Ulcerative Colitis: Physical GVB Restoration and Anti-Inflammatory IEB Regeneration

Weijian Cheng[1], Yixi Zhu[1], Miaoxizi Luo[1], Xiao Wang[3], Quanlong Chen[1], Siyao Li[1], Jing Xian[1], Meng Xiao[1], Licheng Liu[1], Yuanyuan Wang[1], Chaomei Fu[1], Ruibing Wang[2], Qian Cheng[1*], and Jinming Zhang[1*]*

1. State Key Laboratory of Southwestern Chinese Medicine Resources, School of Pharmacy, Chengdu University of Traditional Chinese Medicine, Chengdu, 611137, China.
2. State Key Laboratory of Quality Research in Chinese Medicine, Institute of Chinese Medical Sciences, University of Macau, Taipa, Macau SAR 999078, China.
3. Faculty of Pharmacy and Food, Southwest Minzu University, Chengdu & Key Laboratory of Research and Application of Ethnic Medicine Processing and Preparation on the Qinghai Tibet Plateau, 610225, China

* Corresponding to: Jinming Zhang (email: cdutcmzjm@126.com); Qian Cheng (email: cheng1220cn@163.com); Ruibing Wang (email: rwang@um.edu.mo)

Supplementary Information

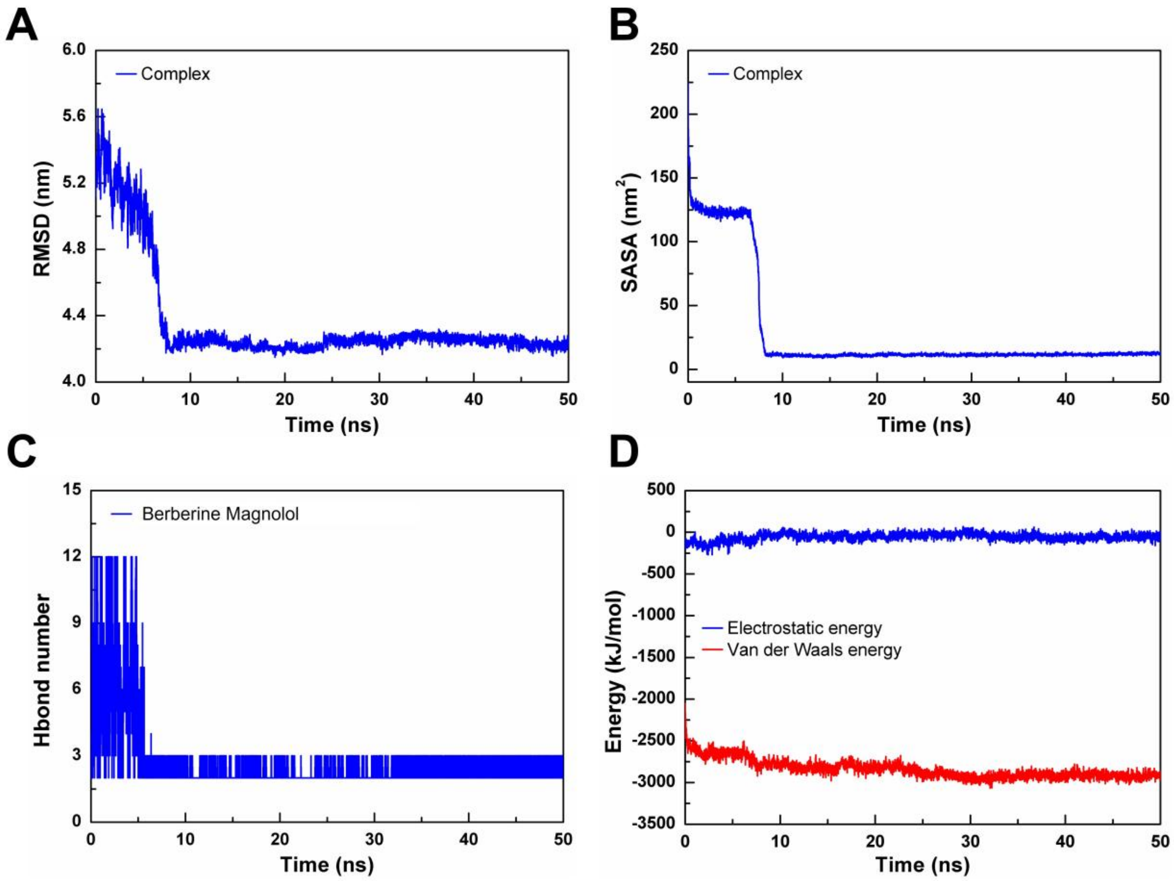

Figure S1. Molecular modeling of BM NPs. (A) RMSD of the aggregate of BM. (B) SASA of the aggregate of BM. (C) Quantitative analysis of intermolecular hydrogen bonding in BM. (D) Interaction energy analysis in BM.

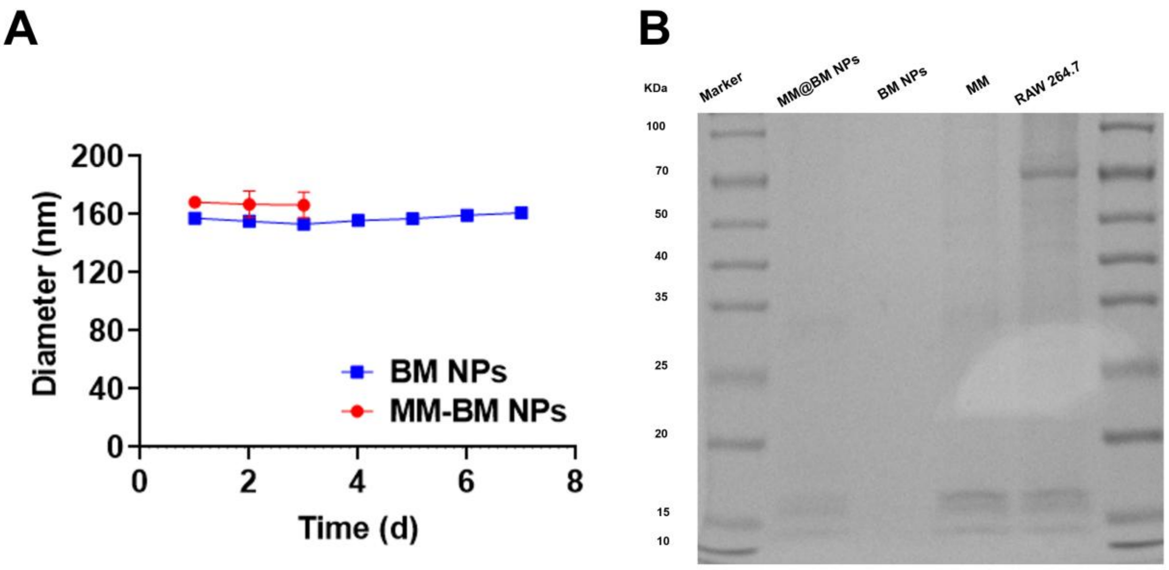

Figure S2. Stability and SDS gel electrophoresis of MM-BM NPs. (A) Diameter changes of MM-BM NPs and BM NPs determined by DLS. (B) Western blot analysis of protein bands of MM-BM NPs.

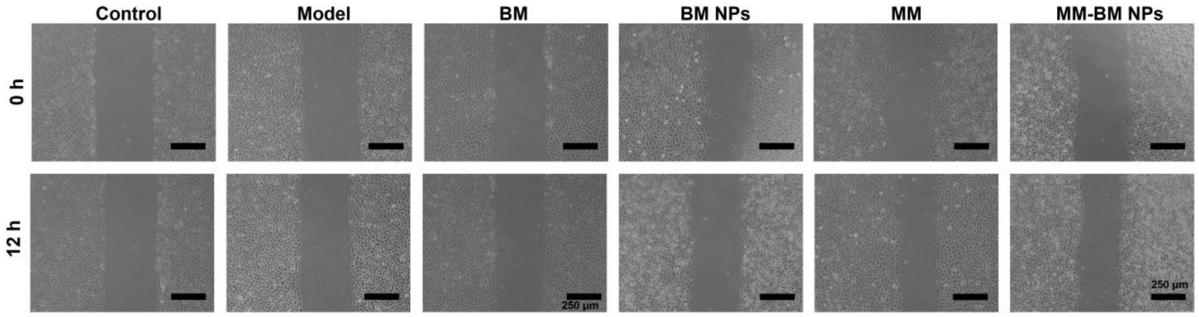

Figure S3. Representative images of HUVECs migration after 12h of treatment with different preparations

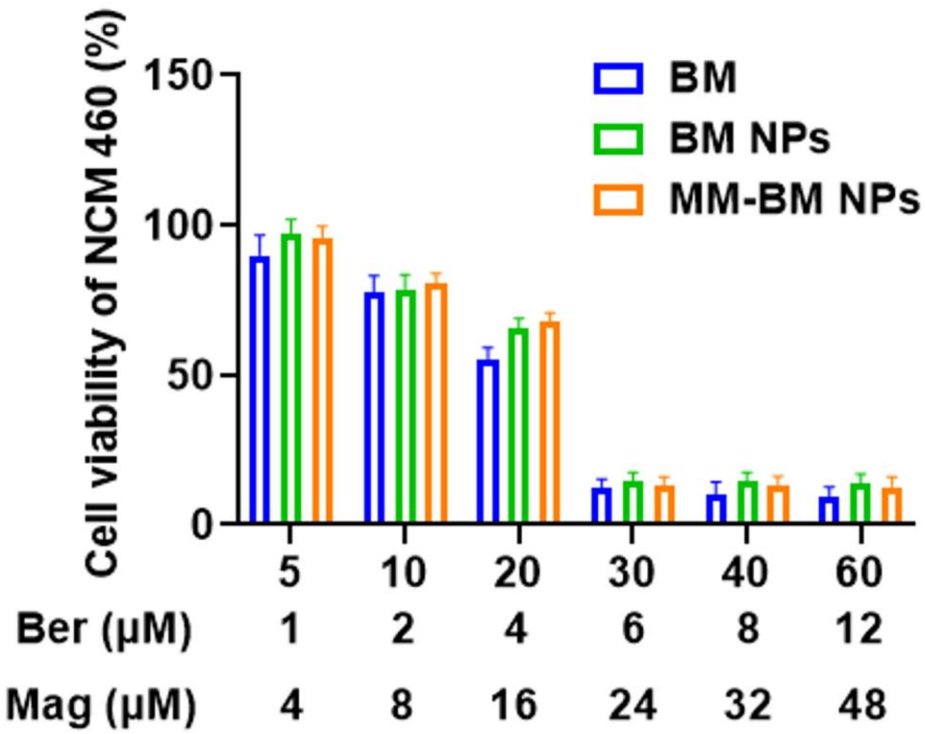

Figure S4. Effect of co-incubation of NCM 460 cells for 24 h on cell viability based on CCK8 assay.

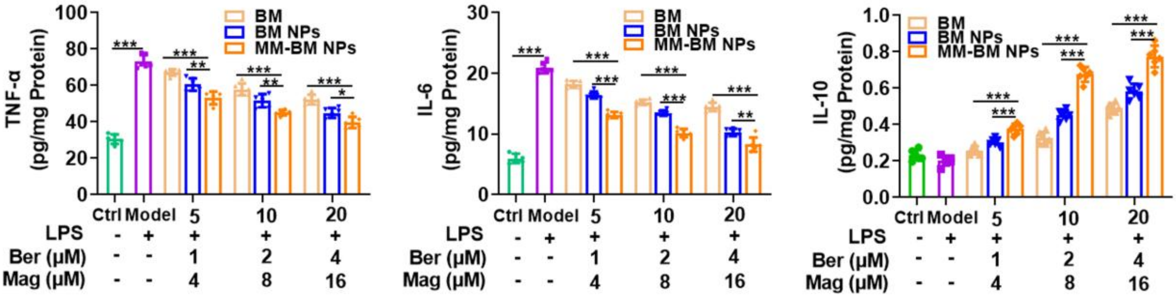

Figure S5. Effects of different preparations on the amounts of proinflammatory (TNF-α and IL-6) and anti-inflammatory (IL-10) factors produced by NCM 460 cells. Data are mean ± SD. *$p < 0.05$, **$p < 0.01$, ***$p < 0.001$.

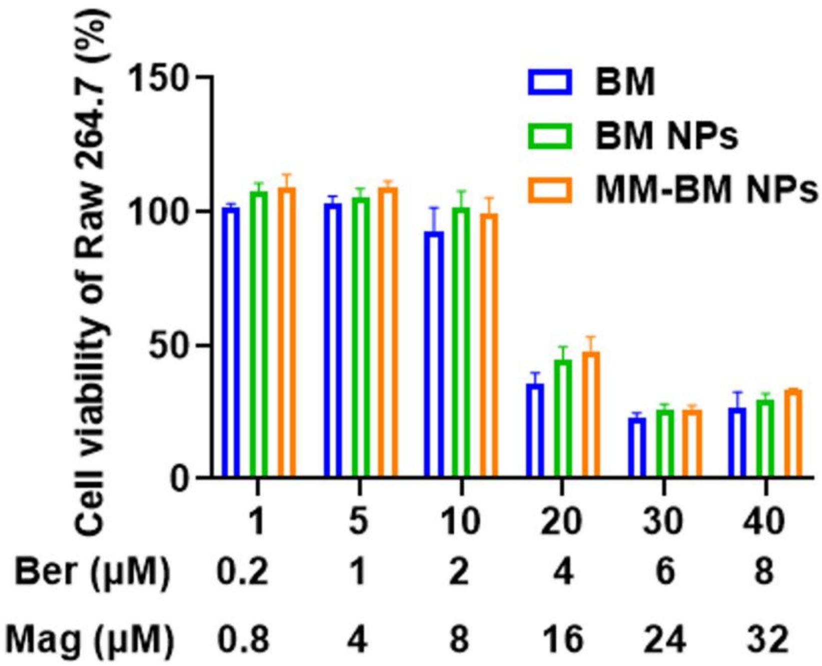

Figure S6. Effect of co-incubation of RAW 264.7 cells for 24 h on cell viability based on CCK8 assay.

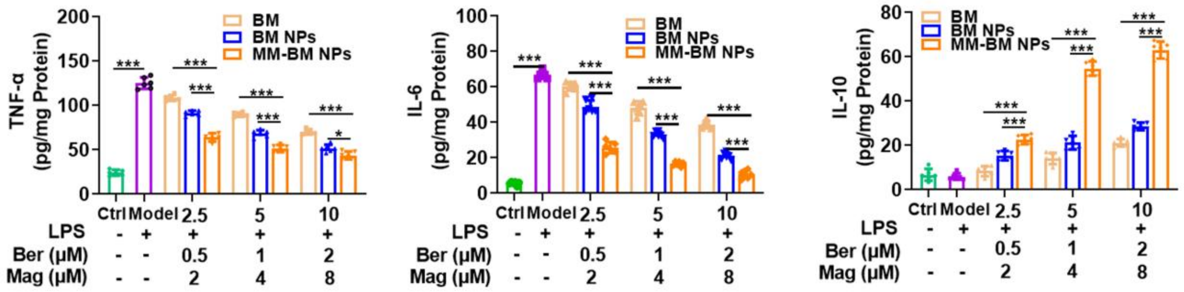

Figure S7. Effects of different preparations on the amounts of proinflammatory (TNF-α and IL-6) and anti-inflammatory (IL-10) factors produced by RAW 264.7 cells. Data are mean ± SD. *$p < 0.05$, **$p < 0.01$, ***$p < 0.001$.

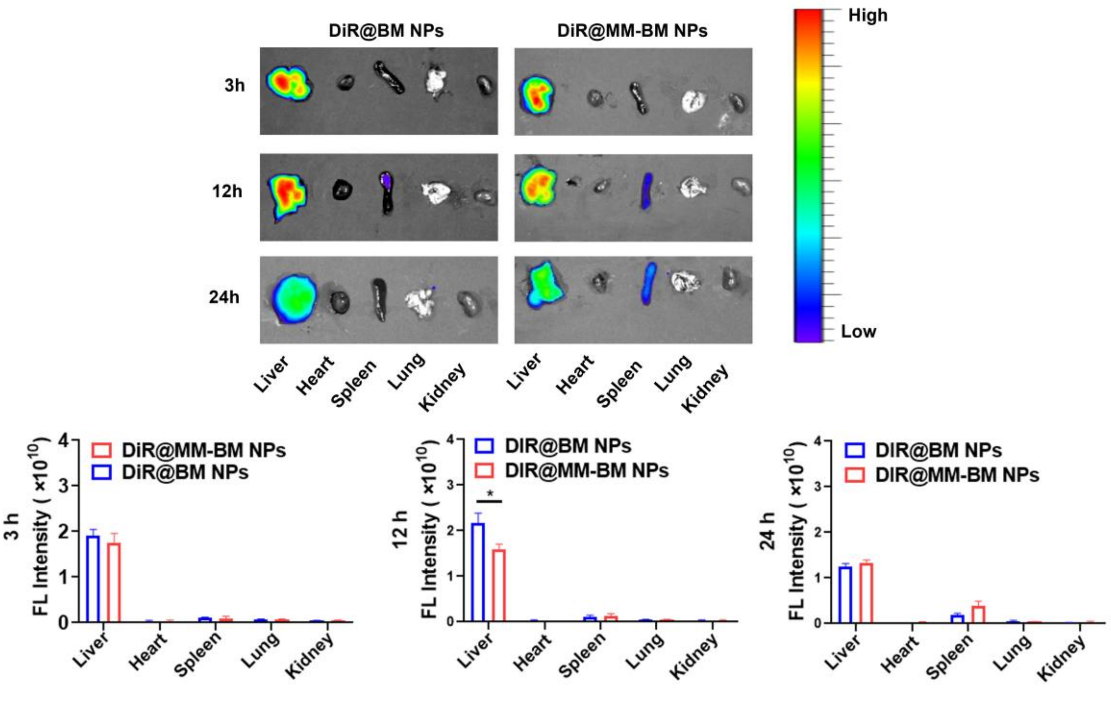

Figure S8. The fluorescence distribution of main visceral organs in mice and the quantitative statistical histogram of fluorescence intensity of different organs. Data are mean ± SD. *$p < 0.05$, **$p < 0.01$, ***$p < 0.001$.

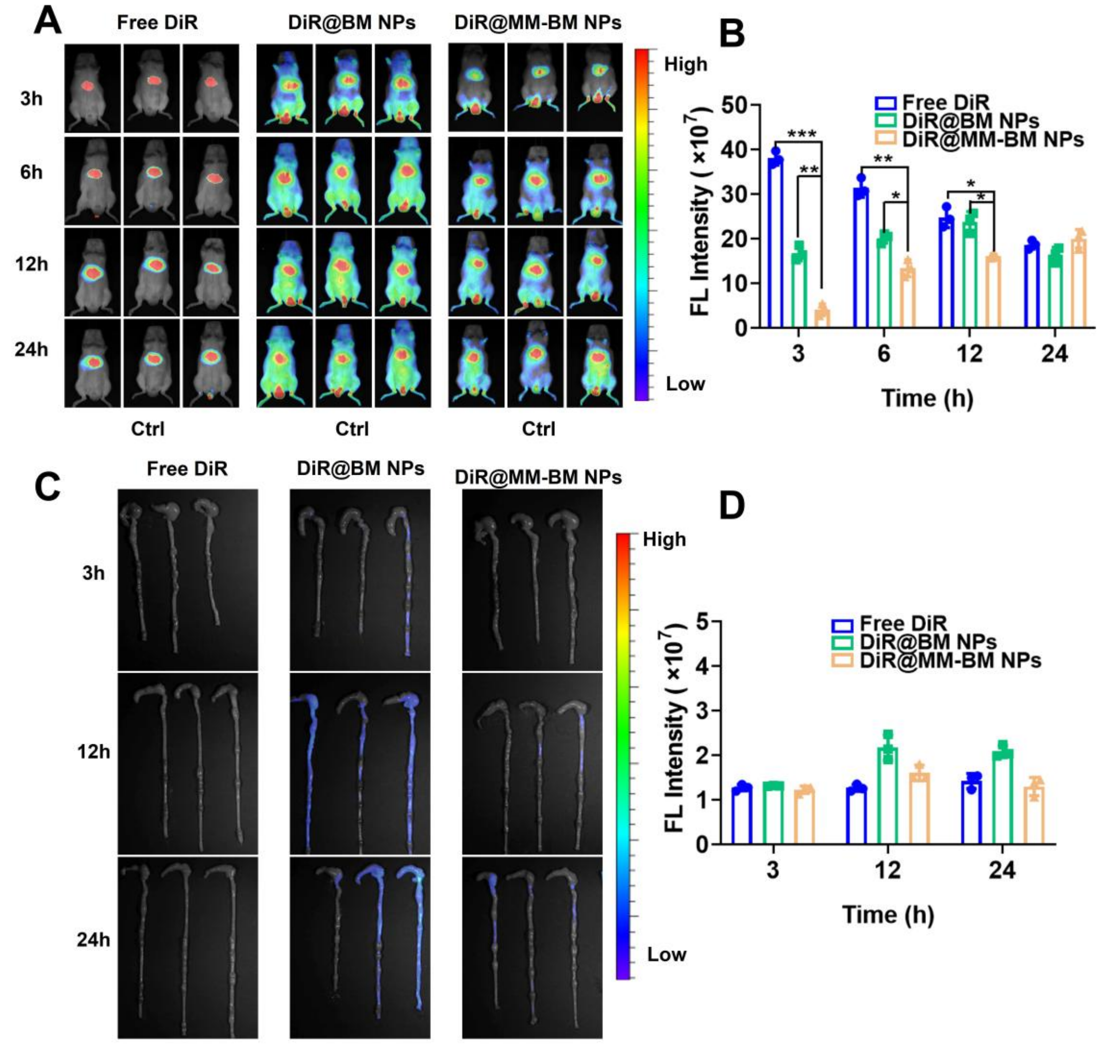

Figure S9. In vivo distribution of MM-BM NPs in healthy mice. (A) Fluorescence images of healthy mice after intravenous administration of free DiR, DiR@BM NPs, and DiR@MM-BM NPs at 3, 6, 12, and 24 h. (B) Histogram of the fluorescent signal analysis at 3, 6, 12, and 24 h. (C) Fluorescence images of colon tissues after intravenous different formulations at 3, 12, and 24 h. (D) Histogram of the fluorescent signal analysis of colon tissues. Note: Data are mean ± SD. *$p < 0.05$, **$p < 0.01$, ***$p < 0.001$. This experiment was analyzed by IVIS Spectrum (VisionWorks, Analytik Jena, US).

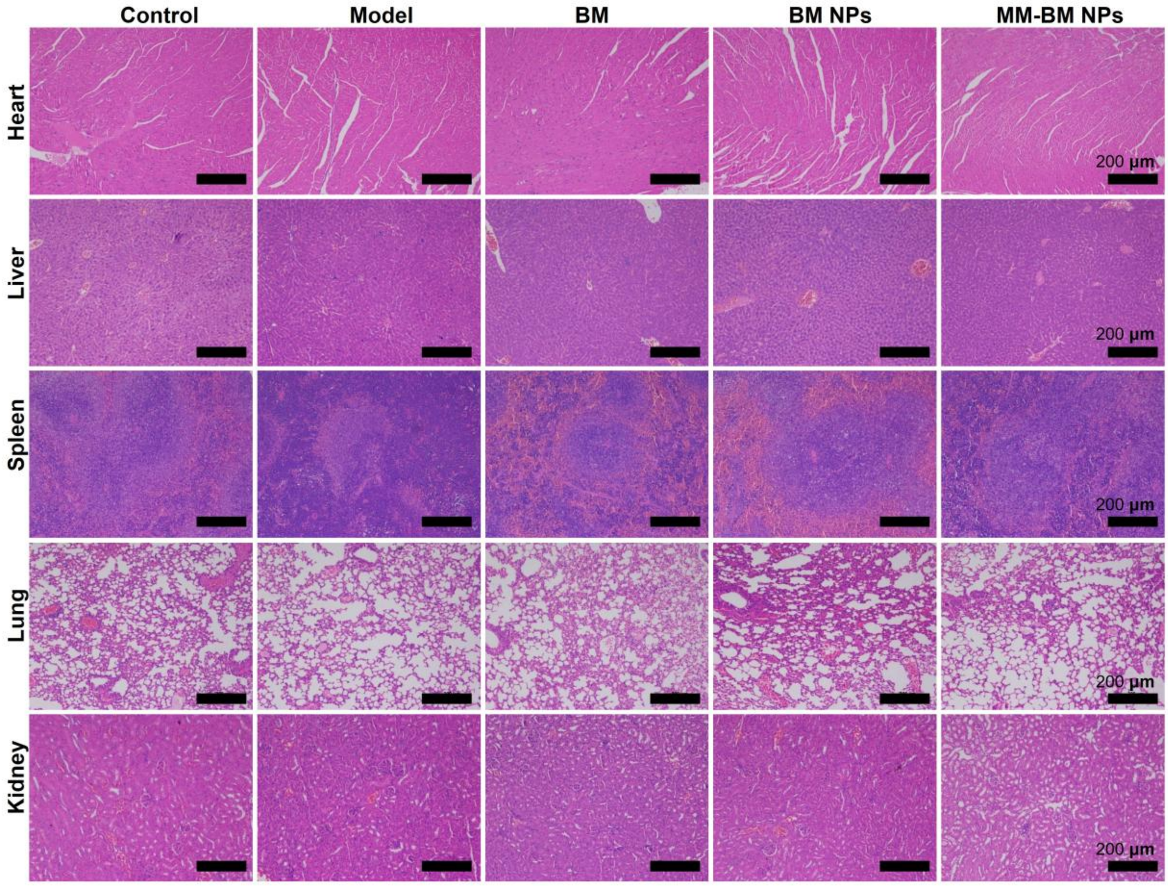

Figure S10. The scanning images of H&E staining of main internal organs, including the heart, liver, spleen, lungs, and kidneys of mice.

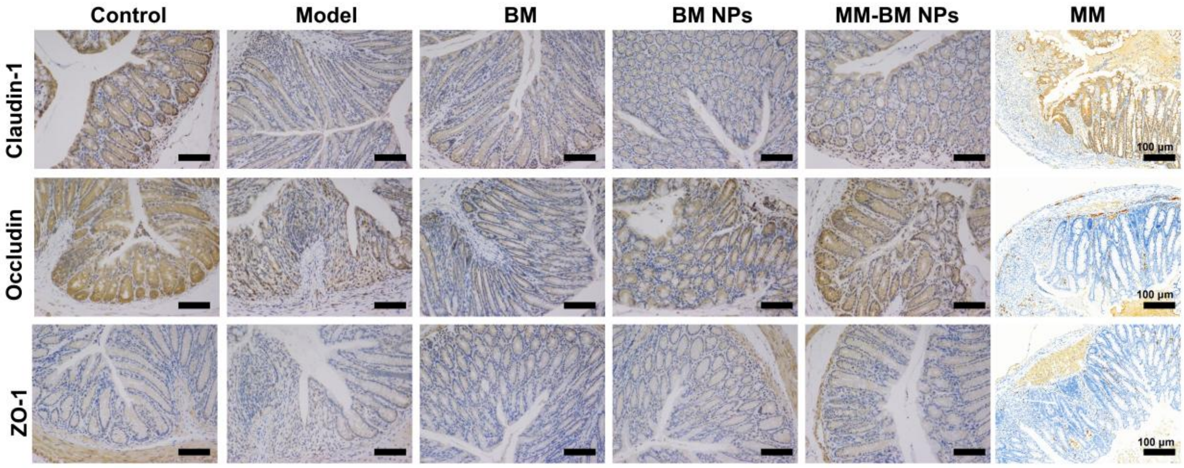

Figure S11. Immunohistochemical analysis of ZO-1, Occludin, and Claudin-1.

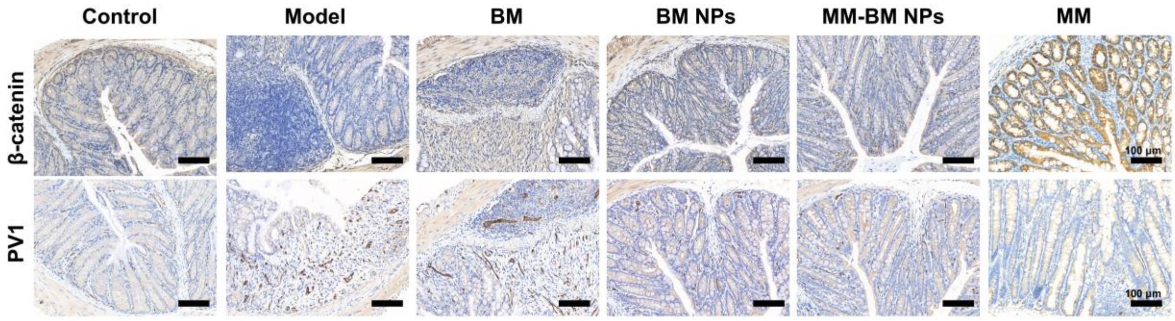

Figure S12. Immunohistochemical analysis of β-catenin and PV1.

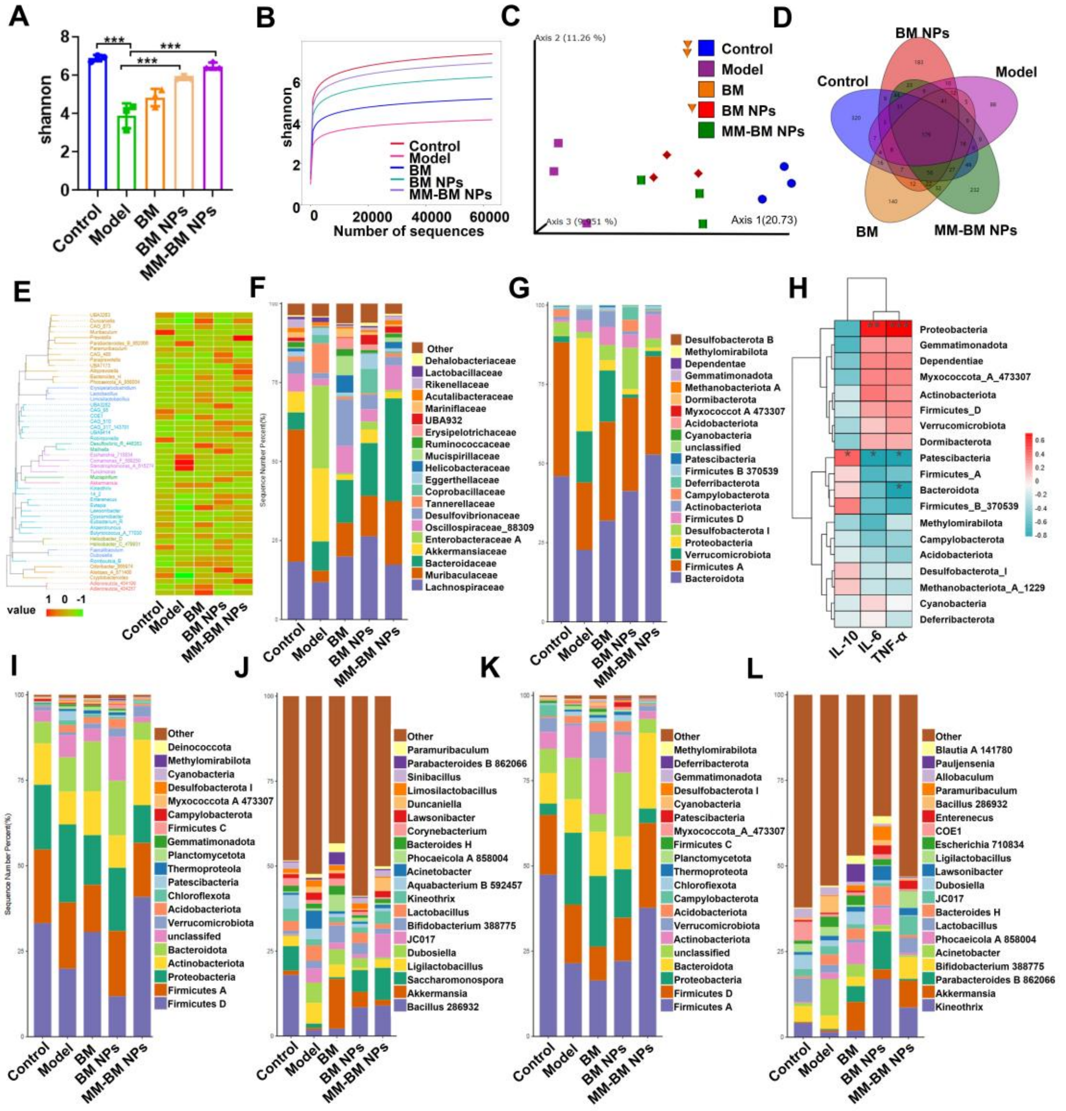

Figure S13. Regulation of intestinal flora and intervention of intestinal flora translocation by MM-BM NPs in mice. (A) Shannon's alpha diversity index of intestinal flora. (B) 2D-PCoA analysis of intestinal flora. (C) α-diversity dilution curve for each group. (D) Venn diagram of intestinal flora. (E) Phylogenetic tree and heat map of abundance distribution of intestinal flora. (F) Relative abundance of the top 20 species at the phylum level of intestinal flora. (G) Relative abundance of the top 20 species at the family level of intestinal flora. (H) Correlation analysis of 20 flora with phylum levels of pro-inflammatory and anti-inflammatory cytokine of intestinal flora. Red and blue colors represent positive and negative associations, respectively. The different levels of colors represent the degree of correlation. (I) Relative abundance of top 20 species at phylum in the livers of each group. (J)

Relative abundance of top 20 species at family level in the livers. (K) Relative abundance of top 20 species at phylum in the pancreas of each group. (L) Relative abundance of top 20 species at family level in the pancreas of each group. Data are mean ± SD. *$p < 0.05$, **$p < 0.01$, ***$p < 0.001$.

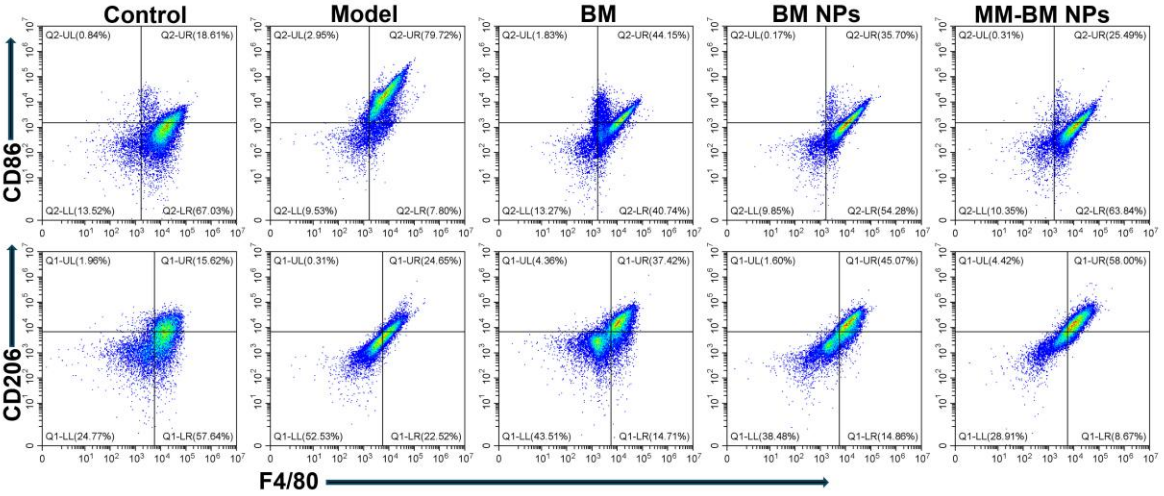

Figure S14. Flow cytometry results of CD86 and CD206 expressions in colon incubated with each group.

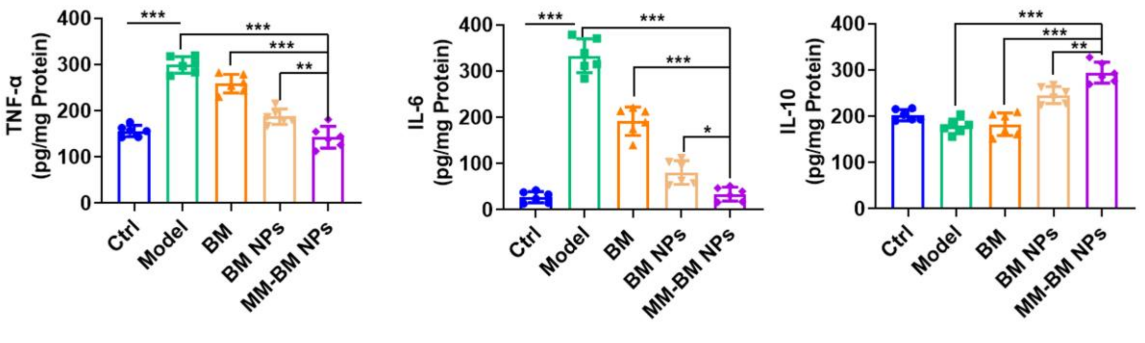

Figure S15. Histogram analysis of different preparations on the changes of TNF-α, IL-6, and IL-10 in the colon. Data are mean ± SD. *$p < 0.05$, **$p < 0.01$, ***$p < 0.001$.
